# Pervasive Indigenous and local knowledge of tropical wild species

**DOI:** 10.1007/s13280-024-02100-w

**Published:** 2024-12-14

**Authors:** Yoshito Takasaki, Oliver T. Coomes, Christian Abizaid

**Affiliations:** 1 Graduate School of Economics, University of Tokyo, 7-3-1 Hongo, Bunkyo-ku, Tokyo 113-0033 Japan; 2 Department of Geography, McGill University, 805 Sherbrooke Street West, Montreal, QC H3A 0B9 Canada; 3 Department of Geography & Planning and School of the Environment, University of Toronto, 100 St. George St, Toronto, ON M5S 3G3 Canada

**Keywords:** Amazonia, ILK concordance, ILK pervasiveness, Indigenous and local knowledge (ILK)

## Abstract

The promise of Indigenous and local knowledge (ILK) for conservation policy depends on how pervasively ILK is held among local people. In the Peruvian Amazon, we conducted a landscape-scale concordance analysis between (1) ILK for game, timber, and fish species collected by the largest representative ILK survey as yet undertaken in tropical forests, and (2) remotely sensed land cover as proxies for species habitat. From our survey among 4000 households in 235 communities, we find that concordant ILK is highly pervasive across gender, age, place of origin, and social status, irrespective of species and people’s indigeneity. Resource users possess more concordant knowledge than nonusers for timber and fish, not game. Concordance between ILK for fish and remote sensing is associated with cooperative forest clearing in shifting cultivation—an informal community institution in which forest peoples engage with nature. Our findings point to the promise of ILK for large-scale tropical conservation.

**Supplementary Information:**

The online version contains supplementary material available at 10.1007/s13280-024-02100-w.

## Introduction

Indigenous peoples and local communities (IPLCs), in whose territories lie much of the world’s biodiversity (Garnett et al. 2018), possess rich place-based ecological knowledge and a deep relationship with nature (Mistry and Berardi 2016; Brondízio et al. 2021). Indigenous and local knowledge (ILK) constitutes various systems of knowledge, practice, and beliefs (Berkes 2017). As an important part of the culture and identity of IPLCs, ILK is valuable in and of itself. At a more practical level, ILK can offer ways to understand and better address the global challenges of biodiversity conservation and sustainability while enhancing inclusiveness, empowerment, equity, and legitimacy (Brondizio and Le Tourneau 2016; Brondízio et al. 2021). Specifically, ILK can complement western science through the coproduction of knowledge (Roue and Nakashima 2018; Thompson et al. 2020; Brondízio et al. 2021), in formulating multiple evidence bases (Tengö et al. 2014, 2017), and in addressing scale mismatching in socio-ecological systems (Cumming et al. 2006; Herse et al. 2020). Moreover, ILK can play a key role in understanding nature’s contributions to people’s quality of life (Pascual et al. 2017; Díaz et al. 2018; Brauman et al. 2020). The importance of mainstreaming ILK in global environmental policy is embodied in the Convention on Biological Diversity (CBD) and ILK has been prominently recognized by the Intergovernmental Science-Policy Platform on Biodiversity and Ecosystem Services (IPBES) (Díaz et al. 2015, 2019; Tengö et al. 2017; Brondízio et al. 2019; Hill et al. 2020; McElwee et al. 2020; Reyes-García et al. 2022).

In this paper, we examine an important question that has not yet been explored in a systematic way: how widely is ILK held among IPLCs? The pervasiveness of ILK, which is critical to better understand knowledge systems, conditions its promise for conservation policy and sustainability. Generally, two views coexist in the literature: (1) ILK is pervasive among IPLCs as a cultural feature and marker; and, (2) ILK is possessed predominantly by certain individuals or groups within IPLCs, such as elders or expert hunters/fisherfolk. The former view underlies the recognition of ILK in policy frameworks and is predominant among social scientists. The latter view is evident in studies, often conducted by conservation scientists and practitioners (Aswani et al. 2018; Joa et al. 2018), which typically target a small number of communities according to policy goals (e.g., protection of certain species) and “knowledgeable” informants within those communities according to specific attributes and/or using reference, snowball sampling, and/or screening (Davis and Wagner 2003; Aswani et al. 2018; Joa et al. 2018). Though useful, this latter approach cannot assess how broadly ILK is held beyond targeted communities and informants. Instead, sampling designs that secure “representative” knowledge based on census or random sampling of communities and informants are required, as used in social sciences and development studies (Deaton 1997).

**Fig. 1:**
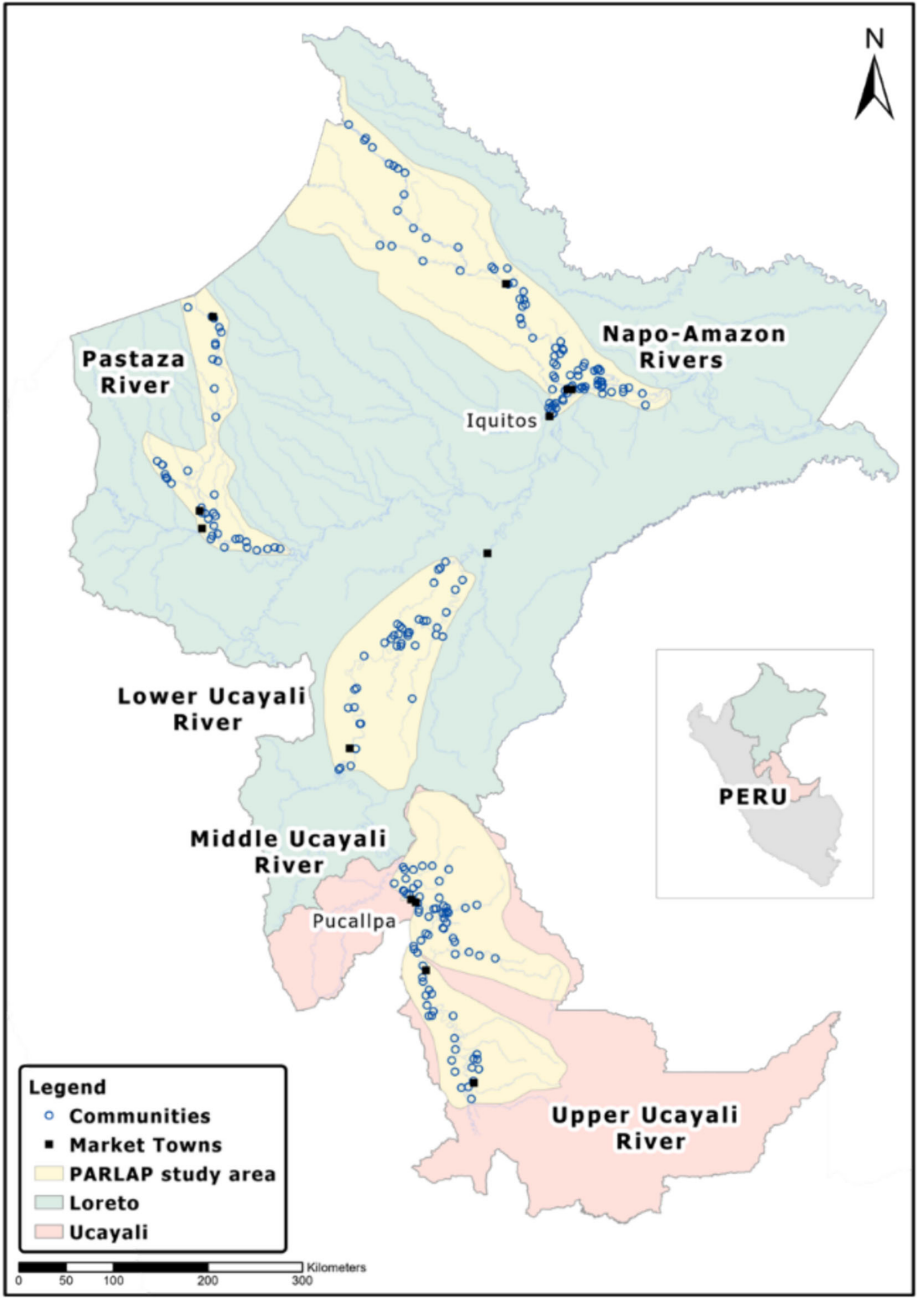
Communities covered in household survey in Peruvian Amazon. The map shows 235 communities covered in the household survey as part of the Peruvian Amazon Rural Livelihoods and Poverty (PARLAP) project conducted (from August 2014 through July 2016) in the Departments of Loreto and Ucayali in Peru

We conducted a survey with close to 4000 randomly selected households in 235 randomly selected communities (henceforth *original sample*) in the Peruvian Amazon—the largest ILK survey as yet undertaken in tropical forests (Fig. 1). This unique sampling design at a landscape scale allowed us to collect ILK representative of both communities and the region. Given their reliance on wild resources for livelihoods, forest peoples are considered to hold considerable knowledge about local wild species. We focused on the most basic component of knowledge—species presence (Berkes et al. 2000)—which although crude, is critical for species conservation. Examining this basic-level ILK is useful and effective because if the pervasiveness of ILK is indeed high among forest peoples, we expect to see such high pervasiveness for this most basic of measures. Our focus lies on assessing the *pervasiveness* of, rather than the *content* of ILK (Cámara-Leret et al. 2014; Cámara-Leret and Dennehy 2019). We do so by leveraging the rich variation in ILK within and across communities based on large-scale representative survey data. Our large-scale approach, on this basic ILK measure, is complementary to conventional approaches that capture ILK more holistically in a small number of communities.

In addition to the matter of pervasiveness of ILK among IPLCs, there persists the unanswered question of concordance between ILK and western scientific knowledge over large scales, especially in tropical forests. Concordance would point to the promise of combining these different knowledge systems for policy formulation at landscape scales. We conducted a statistical concordance analysis at a landscape scale that compares species presence with remotely sensed land cover that captures species habitat (Takasaki et al. 2022). This concordance analysis does not presume relative superiority of remote sensing data or ILK. Although species presence (basic ILK) cannot capture ILK holistically, we consider the pervasiveness of concordant ILK as an emergent property of knowledge systems among IPLCs. As such, we seek to bridge ILK and western scientific knowledge in research practice, which may be critical to advance mainstreaming ILK in policy formulation. This systematic study is a first step in seeking evidence to assess the pervasiveness of concordant ILK at a landscape scale.

## Theoretical framework

### Knowledge, pervasiveness, and concordance

We develop a novel framework to assess the pervasiveness of concordant ILK. We consider *collective knowledge* about community-level information (e.g., species presence in the community) to be generated from *individual knowledge* held by people in the community. Collective knowledge can be distinct across different groups of people in the community (e.g., men vs. women) and how homogeneous such collective knowledge is captures the *pervasiveness of knowledge* in the community. Importantly, the pervasiveness of knowledge does not depend on the *concordance* of collective knowledge, i.e., knowledge may be pervasive but not necessarily concordant with knowledge generated by alternative epistemological approaches (e.g., western science). We consider *pervasiveness of concordant knowledge* (henceforth PCK) as the degree of homogeneity in the concordance of collective knowledge held among different groups of people in the community. Then, PCK is determined by: (1) how widely held collective knowledge is among different groups of people (pervasiveness); and (2) how concordant collective knowledge is within each group (concordance). PCK, as an emergent property of holistic knowledge systems, may be related to informal community institutions, in which people engage with nature, as part of knowledge systems.

### Assessment of prevalence and concordance

Our household survey collected information about the presence of 20 indicator species in three groups (9 game species, 6 species of timber, and 5 species of fish; Table S1) around the community at the time of the survey from heads of households (individual knowledge). We considered the proportion of households in a given community reporting species presence as community-level collective knowledge and constructed indices that capture the overall presence of game, timber, and fish species as discussed below. To assess the pervasiveness of knowledge, we repeated the construction of these ILK measures (collective knowledge) for different groups of respondents discussed below for comparison. Greater similarity (or consensus, Braga‐Pereira et al. 2024) in the ILK measure across respondent groups points to more pervasive ILK among people.

Our landscape-scale concordance analysis compared these ILK measures with two remotely sensed land cover measures across communities: (1) forest cover as a proxy for terrestrial habitat for game and timber; and (2) non-main channel open water as a proxy for floodplain aquatic habitat for fish (Skole and Tucker 1993; Hanski 2011). If ILK and remotely sensed land cover effectively capture species presence and habitat, respectively (Turner et al. 2003), then the two measures should be positively correlated. This first-order concordance would point to the promise of combining ILK and remote sensing for large-scale monitoring (Turner et al. 2003; Pettorelli et al. 2014a, 2014b; Turner 2014) as found in earlier work on the concordance (but not pervasiveness) of ILK based on the original sample (Takasaki et al. 2022). This concordance analysis overcomes the lack of ground-level data over extensive areas using conventional in situ monitoring methods, such as line-transects, camera trapping, and harvest censuses used for small-scale assessment (Anadón et al. 2009; Polfus et al. 2014; Fernández-Llamazares et al. 2016; Camino et al. 2020). Our approach builds on the consistency and expansiveness of remote sensing measures without presuming their superiority relative to ILK. To assess PCK, we repeated this concordance analysis for different groups of respondents and compared them. Greater similarity in the concordance across respondent groups would indicate that concordant ILK is more widely held among people.

## Materials and methods

### Study context

Our study area was located in the Departments of Loreto and Ucayali in Peru (Fig. 1), where much of the land lying below 200 m asl is covered by humid tropical forest in a landscape dominated by rivers and wetlands (Toivonen et al. 2007). The estimated population of Loreto and Ucayali in 2017 was about 1 380 000 with 73% living in urban areas (INEI 2018). The two primary cities of Iquitos and Pucallpa serve as major urban markets and administrative centers, smaller towns function as district capitals and secondary markets, and between the towns, small communities with limited access to public services line the rivers. Rural populations self-identify predominantly as Indigenous peoples or Mestizos. Mestizos (locally known as *ribereños*) are descendants over many generations of Iberian and Indigenous peoples living in the region (Chibnik 1991). Forest peoples are among the poorest economically in the country. Forest peoples engage in agriculture, fishing, hunting, timber harvesting, nontimber forest product gathering, and small livestock raising for subsistence and cash earnings, sending produce to market by boat (Chibnik 1994; Kvist et al. 2001; Takasaki et al. 2001). Depending on local resource endowments, livelihood patterns are significantly different across communities as well as among households (Coomes et al. 2016).

### ILK survey and measures

In the Peruvian Amazon Rural Livelihoods and Poverty (PARLAP) project^1^, we selected four major river basins—the Amazon, Napo, Pastaza, and Ucayali (near 120 000 km^2^, or about half the area of the United Kingdom; Fig. 1)—to capture the diversity of ecological conditions, economic activities, history, and indigeneity of its peoples (Coomes et al. 2016). We conducted a community survey, from September 2012 through March 2014, among 919 communities, which we estimate represents 92% of all communities in the study area (i.e., a near census). We conducted a household survey, from August 2014 through July 2016, in randomly sampled 235 communities from the community survey sample using sub-basins, indigeneity, and resource endowments at community establishment at the current site as strata (Fig. 1). The household survey was conducted among all available households if the community consisted of up to 20 households, or randomly sampled 20 households in communities with more than 20 households (mean: 16.7 households per community; Fig. S1). The household survey covered a total of 3929 households. With this sampling design, our community and household samples for the household survey (original sample) are representative in the PARLAP study area.

Twenty indicator species of game, timber, and fish (Table S1) were selected to capture a range of market values as well as cultural preferences and vulnerabilities to harvesting pressure. Game species are those commonly hunted and are classified into three groups: large-bodied mammals, small-bodied mammals, and monkeys. Timber and fish species are grouped into 1st class and 2nd class species by local preferences and market value. More information about these indicator species is provided in earlier work (Takasaki et al. 2022). Lists of indicator species using Spanish common names were presented to the heads of households. Respondents were asked whether each species could be encountered within one day’s travel from the community (by foot for game and timber; by canoe for fish) at the time of the survey. ILK for game species collected from a similar household survey was found to be consistent with results from line-transects and camera trapping in one of the communities in the community survey sample (Zayonc and Coomes 2021). Law enforcement against illegal commercial hunting in the region is limited among rural communities. According to our fieldwork experience in the region since the 1990s, respondents’ misreporting reflecting illegality or conservation concerns is unlikely to be a major concern (Takasaki et al. 2022).

We constructed two sets of ILK measures at the community level (collective knowledge): (1) the proportion of households reporting the presence of each of 20 indicator species in the community, and (2) *z*-score of first factor of principal component analysis on these proportional measures for game, timber, and fish species. *z*-score is equal to (*x—μ*)/*σ*, where *x* is a raw score, *μ* is the mean of *x*, and *σ* is the standard deviation of *x*; *z*-score whose unit is a standard deviation facilitates the interpretation of analysis results. Considerable variations exist in the presence of species reported by respondents in the same community (Fig. S2) and the proportion of communities where individual knowledge was uniform varies across species in a consistent way (Fig. S3). The distributions of the ILK indices are shown in Fig. S4A–C.

### Land cover measures

We used Landsat TM, ETM+ and OLI images for 2015 from USGS Earth Explorer. All images were classified into *forest*, *non-forest*, and *other* classes with CLASlite v 3.2 (Asner et al. 2009), where the *other* class included clouds, cloud shadows, and water (for more details on methods and data, see Takasaki et al. 2022). We used surface water classified from the Landsat Surface Reflectance Tier 1 imagery collection in Google Earth Engine for 2015. The method and data are described in a data descriptor (Kalacska et al. 2022). The resultant surface water was classified into *main channel* and *non-main channel open water*. Non-main channel open water is found off the main channel of river, i.e., lakes, abandoned side-channels, streams, and other forms of standing water on the floodplain, which constitutes critical habitat for freshwater fish in the region (Junk et al. 2007; Arantes et al. 2019; de França Barros et al. 2020). Forest cover and non-main channel open water, respectively, are measured by the proportion of forest on land and non-main channel open water in a 5 km buffer centered on each community. The distributions of forest cover have a thick upper tail and those of non-main channel open water have a very thick lower tail (Fig. S4D, E).

The following caveats are noted. First, although our remotely sensed land cover measures capture overall habitat conditions, they are imprecise at capturing species-specific habitat given the varied habitat requirements of individual species and thus the concordance analysis does not necessarily reflect concordance of ILK across individual species (Takasaki et al. 2022). We therefore assessed whether the patterns found in the comparison of the ILK indices, which focus on the overall status of species groups mitigating this measurement concern at the species level, are robust across corresponding species without making comparisons for individual species. Second, the concordance for terrestrial (game and timber) and aquatic (fish) species using different land cover measures (forest cover and non-main channel open water) may not accurately reflect the difference in the concordance of ILK between these two groups of species; hence, we did not make such a comparison.

### Analysis samples

For a systematic assessment of pervasiveness, designing an effective comparison across different groups of people is critical. For each comparison of groups of respondents, we constructed an *analysis sample* from the original sample focusing on communities with more than one household within each group of respondents so that collective knowledge can be constructed from individual knowledge in each community for each group of respondents. In this way, we compared respondents within the same community to assess collective knowledge about the same community-level information. Put differently, for each analysis sample, the analysis mimics different targeting of respondents according to certain attributes among those randomly selected in the survey.

We considered respondent gender and household resource use (separate engagement in hunting, timber harvesting, and fishing) as key attributes (Table 1), because wild resource harvesting is done primarily by men in our study area (Espinosa 2010) and it is common in practice to consider resource users as eligible informants (Hitomi and Loring 2018; Joa et al. 2018). Conservation scientists and practitioners typically select main resource users as key informants. We identified main users in terms of harvest intensity and specialization in wild resource harvesting (Table S2). We also considered age (51% of sampled households are in the category "old", using median age (42 years) of respondents as a cutoff), place of origin (55% are from the community in which they reside), and leadership (leader: 31%) as additional attributes, because it is also common in practice to select elders, those born in the community studied, and/or community leaders as informants (Hitomi and Loring 2018; Joa et al. 2018). For gender, age, place of origin, leadership, and kinship (discussed below), the analysis sample for each attribute is common across species. For resource use, harvest intensity, and specialization, the analysis sample for each attribute is different across game, timber, and fish species, because household participation in hunting, timber harvesting, and fishing varies across communities with distinct resource endowments.

**Table 1:** Definition and characteristics of gender, resource use, and kin network analysis samples

	Definition of analysis samples	Summary statistics						
		Original sample		Analysis sample				
		Proportion	No. households	Proportion	No. households	No. Communities	Mean no. households per community	
	(1)	(2)	(3)	(4)	(5)	(6)	(7)	(8)
All (original sample)			3929			235	16.7	
Respondent gender	Male vs. female vs. male and female, depending on whether only male or female respondent reported individually, or they reported jointly (three categories)	0.40	3925	0.36	3084	168	6.6	
Male								
Female		0.22	3925	0.25	3084	168	4.6	
Male and female		0.39	3925	0.39	3084	168	7.1	
Household resource use	Users vs. nonusers, depending on whether the household engaged in hunting, timber harvesting, or fishing	Users		Users			Users	Nonusers
Game		0.28	3929	0.37	2689	154	6.4	11.0
Timber		0.16	3929	0.27	2159	120	4.8	13.2
Fish		0.85	3929	0.74	2041	112	13.5	4.8
Household kin network	Male head-yes vs. male head-no vs. female head-yes vs. female head-no, depending on whether male/female head had any male kin (brother, father, or uncle)/female kin (sister, mother, or aunt) in the surveyed community	Yes		Yes			Yes	No
Male head's male kin		0.56	3920	0.57	3634	205	10.1	7.6
Female head's male kin		0.50	3913	0.51	3627	205	9.0	8.7
Male head's male kin		0.50	3920	0.51	3634	205	9.1	8.7
Female head's male kin		0.47	3913	0.48	3627	205	8.5	9.2

The proportion of each attribute across households (along with household sample size) is in column 2 for the original sample of the household survey and column 4 for corresponding analysis samples. Household sample size for some attributes is slightly smaller than 3929 due to missing values. For each analysis sample, community sample size is in column 6 and the mean number of households per community in each respondent type is in columns 7 and 8

The number of sampled communities varies across different analysis samples, and the sample size of households per community in each group of respondents in each analysis sample varies across respondent groups and communities (Table 1 and Table S2). In the gender analysis sample, female respondents (25%) were less common than male respondents (36%) and joint male and female respondents (39%) (means: 4.6 vs. 6.6 and 7.1 per community; Fig. S1A–C). In the resource use analysis sample, the comparison of household sample size between respondent groups depends on how commonly households participated in resource use: for hunting and timber harvesting with low participation (37% and 27%), users (28% and 16%, respectively) were less common than nonusers; for fishing with high participation (74%), the converse holds true (85% users) (Fig. S1E–G, I–N). The proportion of communities where individual knowledge was uniform varies across species in the gender and resource use analysis samples and uniformity is more common for a small group of respondents in each analysis sample, as expected (Fig. S3).

### Analytical framework

We constructed the ILK measures (proportional measures and indices) for each group of respondents in each analysis sample and then compared the ILK measures and their concordance with land cover across respondent groups in each analysis sample. The assessment of the pervasiveness of knowledge was done by the comparison of means (*t* test) and distributions (Kolmogorov–Smirnov test) and the correlation (Pearson’s correlation coefficients) of the ILK measures across respondent groups. The concordance is measured by the correlation (Pearson’s correlation coefficients) of the ILK and land cover measures, a multi-level model considering spatial clusters, and a nonparametric analysis using a locally weighted regression of ILK on land cover (Cleveland and Devlin 1988). Statistical analyses were undertaken using STATA™ v15.1.

### Local institutions

We consider local informal institutions in which people engage with nature as being potentially related to PCK, an emergent property of knowledge systems. As an example of such community institutions, we focus on cooperative work parties organized along kin networks (Takasaki et al. 2014; Abizaid et al. 2015, 2018) to clear forest using simple tools such as axes and machetes in shifting cultivation, which is a ubiquitous local practice in tropical forests (Heinimann et al. 2017). In the Peruvian Amazon, a household hosting a cooperative labor event for forest clearing usually recruits kin and neighbors to work in its plot for a day or less and offers manioc beer (*masato*) and food, typically fish, to the guests in exchange (Abizaid et al. 2015). According to the community survey, depending on hours worked and food/drink provided, a cooperative labor event took five forms: *minga* (a full day of work with food and drink, 77%), *media-minga* (a half day of work with food and drink, 20%), *mañaneo* (a half day of work with drink and without food, 81%), *ayuda* (small work without food or drink, 11%), and other (unspecified, 14%), where the percentage of communities employing each form at the time of the survey in the original sample is shown in parentheses. We considered communities employing the most or second most intensive combinations—only *minga* (11%) or only *minga* and *media-minga* or *mañaneo*, not others (49%)—as high cooperative labor intensity (60%) and the remaining communities as low intensity. To examine the association of PCK with cooperative forest clearing, we repeated the assessment of PCK among these two types of communities separately. Kin relations can underlie social interactions and local informal institutions such as cooperative forest clearing among forest peoples. We also conducted the assessment of PCK according to kin networks in the community fully distinguished by the gender of household heads and kin groups (Table 1).

## Results

### Community heterogeneity across analysis samples

The assessment of prevalence is based on the comparison across respondent groups in each analysis sample. It is not informative (and could be misleading) to compare analysis samples which consist of different communities as well as households (Table 1 and Table S2). To highlight this point, we first compared the distributions of the ILK indices and land cover measures between the gender/resource use analysis sample and the remaining sample in the original sample (Fig. S4). Some distributions are significantly different across these samples (Kolmogorov–Smirnov tests). This is especially so for the analysis samples constructed according to resource use which are dependent on local resource endowments. Specifically, game and timber species were more common and forest cover was higher in the corresponding analysis samples which exclude communities with limited or null participation. Fish species were less common in the analysis sample which excludes communities with uniform or near uniform participation, though there existed little difference in non-main channel open water. Correspondingly, communities in the resource use analysis samples for game and timber were less common near cities, and the converse holds true for fish; such spatial patterns are not observed for communities in the gender analysis sample (Fig. S5). As such, different patterns across analysis samples could be driven by community heterogeneity.

### Pervasiveness of knowledge

**Fig. 2:**
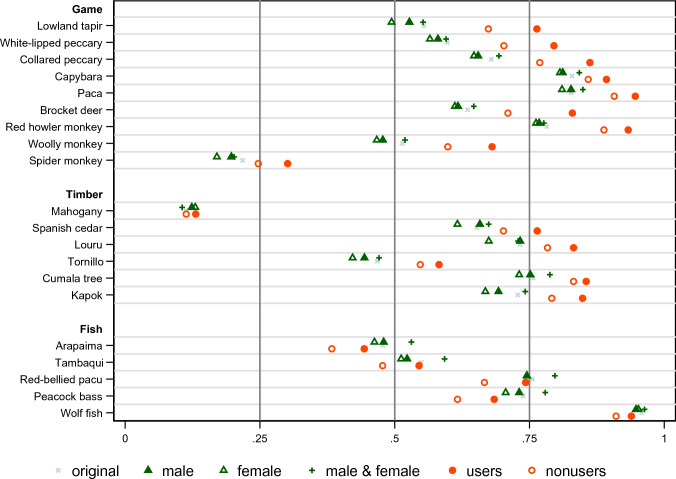
Proportion of presence of species across communities by respondent gender and household resource use. The means of the proportion of households in the community reporting the presence of each of 20 indicator species (i.e., Indigenous and local knowledge, ILK) across communities are shown for each sample. Colors represent different samples. The samples are the original sample of the household survey; male, female, and male and female respondents in the gender analysis sample; and resource users and nonusers in the resource use analysis samples for game, timber, and fish. See Table 1 for the definitions of the analysis samples and respondent groups, and their summary statistics

Overall species presence in the original sample and for each respondent group in the gender and resource use analysis samples is presented in Fig. 2, which shows the means of the proportion of households in the community reporting species presence (i.e., ILK as collective knowledge) across communities (the equality of the means across respondent groups is tested in Table S3). Species presence varies considerably from 11% (mahogany, 1st class timber) to 96% (wolf fish, 2nd class fish) in the original sample. Whereas the patterns of species presence were similar among male and female respondents when they reported separately, the presence of many species was more commonly reported when male and female respondents reported jointly. Respondents in households engaged in resource use reported the presence of corresponding species more commonly than those without users, and the difference between resource users and nonusers was mostly greater than the difference across the three respondent groups according to gender. The comparison of the distributions of the ILK measures (both indices and species) yields consistent and sharper results (Figs. S6 and S7): whereas almost none of the distributions are statistically different across the three gender-related respondent groups, almost all distributions are statistically different between resource users and nonusers (Table S3). The ILK measures are also strongly correlated across these two sets of respondent groups (Fig. S8). Hence, whereas people’s collective ILK is highly pervasive across gender lines within communities, it is different between resource users and nonusers.

### Concordance of knowledge

**Fig. 3:**
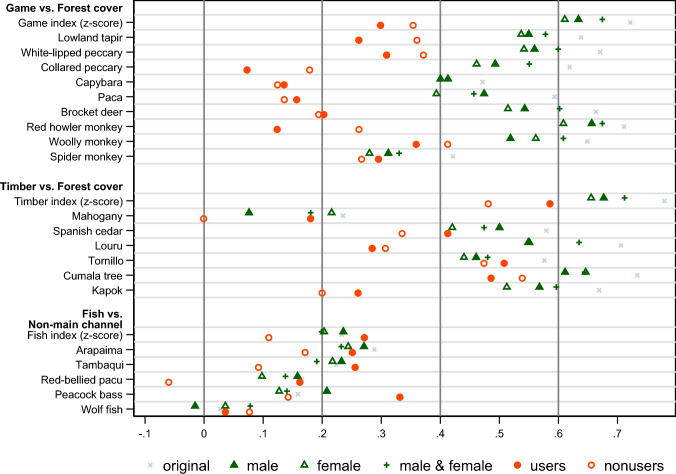
Correlations between ILK and land cover among communities by respondent gender and household resource use. ILK measures are (1) the proportion of households reporting the presence of each of 20 indicator species in the community, and (2) game, timber, and fish indices (*z*-score of first factor of principal component analysis on these proportional measures for game, timber, and fish species). Forest cover and non-main channel open water, respectively, are measured by the proportion of forest on land and non-main channel open water in a 5 km buffer centered on each community. Pearson’s correlation coefficients between the ILK measures and land cover measures among communities are shown for each sample. Colors represent different samples. The samples are the original sample of the household survey; male, female, and male and female respondents in the gender analysis sample; and resource users and nonusers in the resource use analysis samples for game, timber, and fish. See Table 1 for the definitions of the analysis samples and respondent groups, and their summary statistics

Pearson’s correlation coefficients of ILK and land cover measures in the original sample and for each respondent group in the gender and resource use analysis samples are shown in Fig. 3 (*p*-values reported in Table S3). ILK indices and species are positively correlated with land cover at a 5% significance level in the original sample, except for wolf fish. ILK indices are also positively correlated with land cover at a 5% significance level across respondent groups, except for the fish index for nonusers; the results for the corresponding species are largely consistent, though patterns vary by species. These first-order concordance results generally support the concordance of ILK regardless of gender and resource use.

### Pervasiveness of concordant knowledge (PCK)

Whereas the concordance for three indices is similar across the three gender-related respondent groups and the patterns of the corresponding species are similar, the concordance for timber and fish (both indices and species) is stronger among resource users than nonusers (Fig. 3, Table S3). Thus, whereas PCK is high across gender for all species, PCK for timber and fish (not game, as discussed in S1. Robustness check) is higher among resource users than nonusers, indicating that resource users possess more concordant ILK about timber and fish than nonusers. These results are robust to the size of the buffer used to construct land cover measures, the different number of households sampled per community across respondent groups in the analysis sample (Table 1 and Table S2), the covariance within basins according to a multi-level model, and nonparametric model (see S1. Robustness check).

The concordance of ILK is similar between main users (in terms of harvest intensity and specialization) and others, and this is especially so for timber and fish (Fig. S9). Thus, the relatively weak PCK for timber and fish is driven by their participation in, but not by their intensity of, or their degree of specialization in timber harvesting and fishing.

The assessment of PCK according to respondent age, place of origin, and household head leadership status shows high PCK for all species (Figs. S10–S12). When we repeated the assessment of PCK according to gender, age, place of origin, and leadership status among Indigenous and Mestizo communities separately, the results are similar in both types of communities (Figs. S10–S13; see S2. Indigeneity), indicating high PCK for both Indigenous peoples and Mestizos.

### Local institutions

We first compared the distributions of the ILK indices and land cover measures between communities with high and low intensity of cooperative labor defined above (Fig. S14). Forest cover was higher in high intensity communities than in low intensity communities. Correspondingly, timber species, but not game species, were more common in high intensity communities than in low intensity communities. In contrast, the distributions of fish index and non-main channel open water are not significantly different between these two types of communities. Thus, a caveat is needed to interpret the results of the comparison of these two types of communities for game and timber, but not fish, because the comparison could be driven by community heterogeneity.

**Fig. 4:**
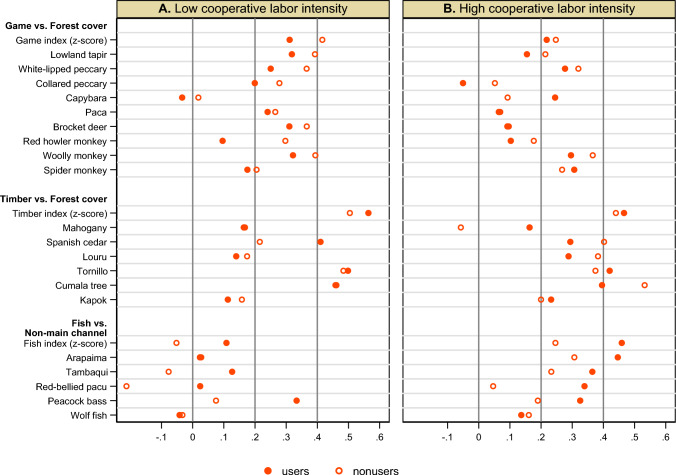
Correlations between ILK and land cover among communities by household resource use and cooperative forest clearing. See the caption to Fig. 3 for ILK and land cover measures. Pearson's correlation coefficients between the ILK measures and land cover measures among communities are shown for each sample. The samples are resource users and nonusers in the resource use analysis samples for game, timber, and fish in communities with low (**A**) and high (**B**) intensity of cooperative forest clearing. See Table 1 for the definitions of the analysis samples and respondent groups, and their summary statistics. High intensity communities employed the most or second most intensive combinations of cooperative labor – only *minga* or only *minga* and *media-minga* or *mañaneo*, not others – and low intensity communities include the remaining forms of cooperative labor (see Local institutions for details)

We repeated the assessment of PCK according to resource use among the two types of communities (Fig. 4; *p*-values reported in Table S4; statistical power in this subsample analysis is relatively weak). For game and timber, the concordance is similar between resource users and nonusers in both high and low intensity communities. For fish, whereas the concordance is stronger among resource users than nonusers in both types of communities, the concordance is statistically significant in high intensity communities for both users and nonusers but not in low intensity communities. At the same time, resource users more commonly reported species presence than nonusers in both high and low intensity communities; as exceptions, users and nonusers reported timber and fish species in a similar way in low intensity communities (Fig. S15;* p*-values reported in Table S4). These results suggest that cooperative forest clearing is related to the concordance of knowledge about fish, but not its pervasiveness.

**Fig. 5:**
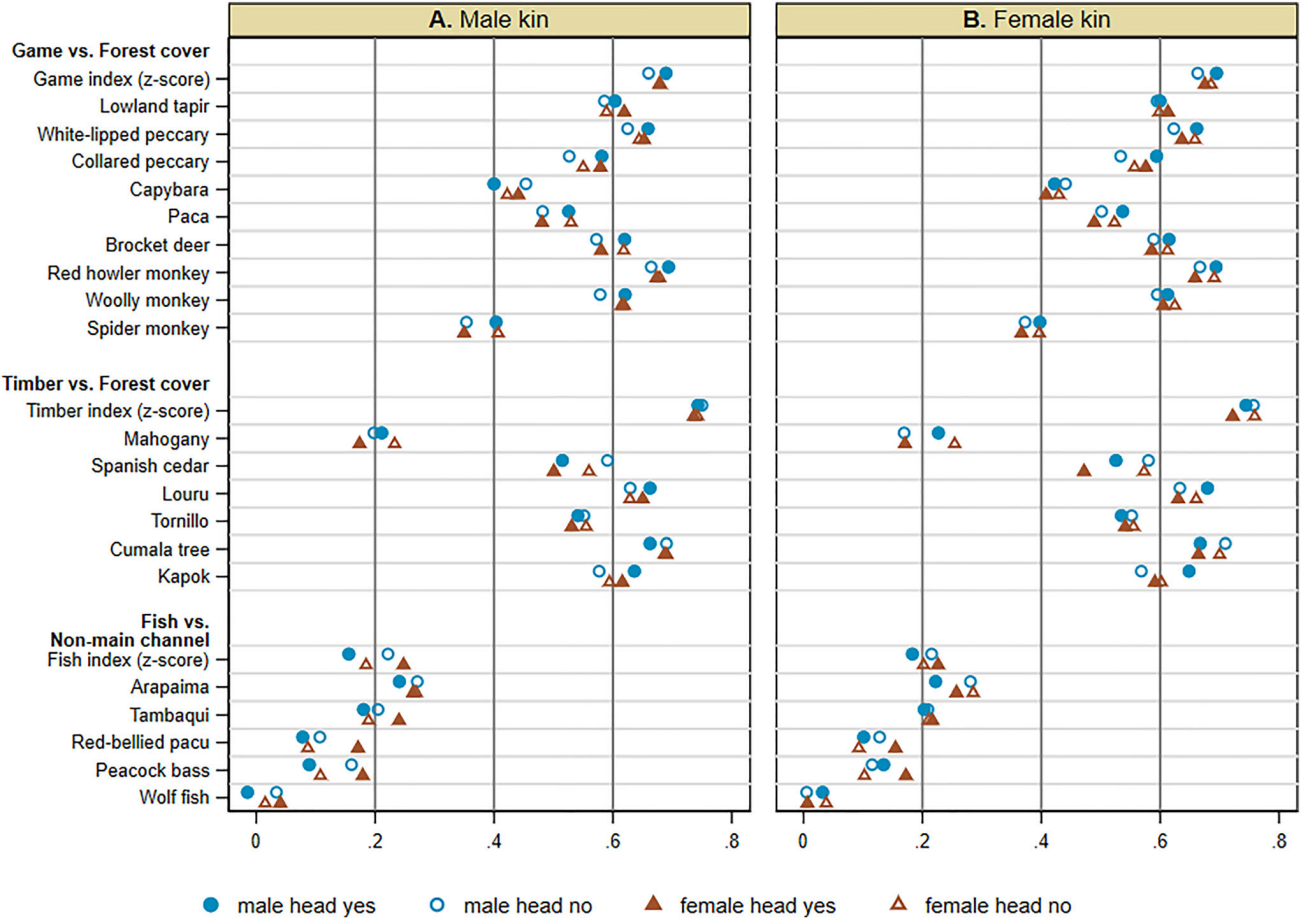
Correlations between ILK and land cover among communities by kin networks. See the caption to Fig. 3 for ILK and land cover measures. Pearson’s correlation coefficients between the ILK measures and land cover measures among communities are shown for each sample. The samples are households with male head with male kin (yes), male head without male kin (no), female head with male kin (yes), and female head without male kin (no) (**A**); households with male head with female kin (yes), male head without female kin (no), female head with female kin (yes), and female head without female kin (no) (**B**) in the kin network analysis sample. See Table 1 for the definitions of the analysis sample and respondent groups, and their summary statistics

Cooperative forest clearing entails at least four important features: kinship, cooperative labor exchange, engagement with nature (forest), and food sharing. Kin relations can underlie broad local institutions as well as social interactions as discussed above. The concordance of ILK is similar across households with and without kin networks (Fig. 5); the pervasiveness of knowledge is also similar (Fig. S16). These results suggest that kin networks per se are not a significant feature, though their roles in cooperative forest clearing can be important. Additional results reported in S3 (Cooperative labor exchange) suggest that cooperative labor exchange per se is also not a significant feature. These results suggest that the combination of people’s engagement with nature and food sharing may be features underling the concordance of knowledge for fish species.

## Discussion

### Interpretations of results

The paucity of available data precludes us from examining how PCK emerged in knowledge systems; however, we can speculate about possible explanations for our findings. Overall, concordant knowledge about the presence of local species is widely held among forest peoples, both Indigenous and non-Indigenous. This is so across gender even though in this setting wild resource harvesting is done mostly by men (cf. Reyes-García et al. 2020). One possible explanation is that knowledge about species presence is transferred from men (who harvest resources) to women within and/or across households (Salpeteur et al. 2016, 2017); another possibility is that women acquire the knowledge through their roles in household work such as preparing, sharing, and selling the harvest (Espinosa 2010). Our data do not allow us to identify which of them are at play. PCK is also high across age, place of origin, and leadership status. The knowledge may be transmitted intergenerationally and among people with different places of origin and social status in the community, and/or widely acquired by people irrespective of age, place of origin, and social status. The high pervasiveness of concordant knowledge in the social sphere is consistent with PCK being neutral to kin networks, which implies that knowledge transfer is not restricted to people with strong social networks.

Resource users possess more concordant knowledge than nonusers for the presence of timber and fish species, but not game; however, the intensity of harvesting is not a distinguishing factor. These patterns may be explained by two factors. First, distinct characteristics of game, timber, and fish species likely imply different needs and feasibility for species status updating. Updating done by resource users is more likely for game than timber and fish, because the status of timber species is visible and less likely to change frequently due to the immobility of timber and, in contrast, the status of fish species is evidently less visible and can change quickly due to the high mobility of fish. Second, knowledge transmission and/or acquisition can occur through food preparation and food sharing among households; distinct from timber, sharing harvested game meat and fish for food is common (Espinosa 2010).

The concordance of knowledge for fish species is associated with cooperative forest clearing. Participation in this local institution in which people engage directly with the forest and share harvested fish might help renew knowledge about the presence of local fish species despite the difficulty in knowing their precise status. Such patterns are consistent with PCK as an emergent property of knowledge systems. Similar patterns are not observed for game or timber species even though forest environments constitute their habitats. This contrast may be related to the distinct composition of resource users and nonusers in a cooperative labor event: for fish, users who can share harvested fish and knowledge about fish species with nonusers are more common than nonusers; the converse holds true for game and timber, and cooperative forest clearing may be commonly organized only among nonusers (Fig. S1F, G, J, K, M, and N). As such, how PCK emerges in knowledge systems may depend on the interplay among livelihoods, social interactions (not necessarily through kin networks), and resource endowments; the emergence of PCK remains for further study.

### Policy implications

Our finding of high PCK on species presence strongly supports the promise of ILK for monitoring, inclusiveness, and scaling up of conservation policy. If concordant ILK is highly pervasive within communities, informant selection can be more flexible, and conservation programs be made more inclusive because informant selection can influence program participation. As such, high PCK can enhance inclusiveness, empowerment, equity, and legitimacy (Danielsen et al. 2014; Brondizio and Le Tourneau 2016). Moreover, more flexible and inclusive participatory local programs could be potentially scaled-up to large areas, which is vital for monitoring for large-scale conservation, especially in biodiverse-rich, data-poor tropical forests (Jones 2011; Luzar et al. 2011; Danielsen et al. 2014; Parry and Peres 2015). For example, a program targeting male hunters as informants may exclude women and other individuals deemed not to engage in hunting, raising important questions around equity (Pfeiffer and Butz 2005; Bechtel 2010; Colfer et al. 2016); with the high PCK across gender, the participation of women as informants can contribute to making conservation programs more inclusive and potentially scalable. A component about basic ILK (such as species presence in the community) could be incorporated into rural surveys, including those targeting women, that cover a wider range of communities than conventional small-scale ILK surveys. Combined with remote sensing, such large-scale ILK-based monitoring can potentially enable participatory conservation at a landscape scale.

## Conclusion

Our landscape-scale assessment of the pervasiveness of concordant ILK, combining representative ILK data and remote sensing in the Peruvian Amazon, offers support for both views held by researchers regarding the pervasiveness of ILK among IPLCs discussed in the Introduction. On one hand, PCK for species presence is high across gender, age, place of origin, and social status among IPLCs, for both Indigenous and non-Indigenous groups and irrespective of species. This novel evidence for high pervasiveness over large spatial scales strongly supports the promise of ILK for conservation policy, especially at a landscape scale, as discussed above. On the other hand, consistent with the view of conservation scientists and practitioners, resource users possess more concordant knowledge about species presence than nonusers for timber and fish, but not game, pointing to the importance of targeting resource users as respondents in order to capture ILK for some resources (timber and fish), as commonly done. More generally, attention should be given to potential differences in PCK across species types; most previous works on ILK in tropical forests tend to focus on one or several species, or a single group of species.

It is crucial to distinguish the pervasiveness of ILK from PCK, which also considers the concordance of ILK. The concordance of ILK for the presence of local species can be associated with people’s experience with nature through local institutions as part of knowledge systems among IPLCs, e.g., for fish, cooperative forest clearing in shifting cultivation. ILK may be maintained and transformed into PCK through the holistic application of knowledge. This insight points to the need for holistic approaches informed by ILK in policy formulation. For example, biodiversity conservation through ILK-based monitoring could be combined with ILK-based sustainable land use in shifting cultivation.

Our initial assessment of PCK opens up promising avenues for future research. First, it would be valuable to extend the assessment of PCK for specific species. To this end, remotely sensed land cover measures need to be developed at finer spatial resolutions specific to species by assessing their concordance with species presence measured by ILK at a landscape scale. Such an approach would contribute to further bridging of complementary ILK and western scientific knowledge. Second, a natural extension of the current work would be to examine ILK beyond species presence, such as ILK on species abundance and health status, or more broadly on ecosystem functions. PCK could vary depending on the complexity of ILK. It may be possible that the more complex ILK, the less concordant and/or pervasive. Understanding how pervasive and concordant different types of ILK are can improve our understanding of knowledge systems and also further inform policy formulation. Third, a better understanding of how PCK emerges in knowledge systems is much needed, and nature-based local institutions as part of knowledge systems would appear to be a promising target of research and policy. Lastly, although our novel approach could be applied to other contexts, application is constrained by the lack of representative ILK data at large scales. Developing an approach to assess the pervasiveness of ILK based on nonrepresentative sample data remains a challenge for future research.

## Supplementary Information

Below is the link to the electronic supplementary material.

Supplementary file1 (PDF 3505 kb)

## Footnotes

1 https://parlap.geog.mcgill.ca
