# Supplementary material for: Pervasive Indigenous and local knowledge of tropical wild species
Source: Ambio. 2024 Dec 14;54(4):680–95. doi: 10.1007/s13280-024-02100-w (PMC11871250; doi:10.1007/s13280-024-02100-w)
Supplement: Supplementary file 1 — Supplementary file1 (PDF 3505 kb) [file 13280_2024_2100_MOESM1_ESM.pdf]

Ambio

Supplementary Information

Title: **Pervasive Indigenous and local knowledge of tropical wild species**

Contents

Supplementary information text
S.1. Robustness check 4
S.2. Indigeneity 5
S.3. Cooperative labor exchange 5

Fig. S1. Histogram plots of household sample size and indigeneity 6
Fig. S2. Histogram plots of proportion of presence of species across communities 7
Fig. S3. Proportion of communities where ILK among respondents was uniform by respondent gender and household resource use 8
Fig. S4. Estimated densities of ILK indices and land cover measures across samples 9
Fig. S5. Communities included in analysis samples 10
Fig. S6. Estimated densities of ILK measures by respondent gender 11
Fig. S7. Estimated densities of ILK measures by household resource use 12
Fig. S8. Correlations of ILK measures among communities across respondent gender and household resource use 13
Fig. S9. Correlations between ILK and land cover among communities by household harvest intensity and specialization 14
Fig. S10. Correlations between ILK and land cover among communities by respondent age and community indigeneity 15
Fig. S11. Correlations between ILK and land cover among communities by respondent place of origin and community indigeneity 16
Fig. S12. Correlations between ILK and land cover among communities by household head leadership in the community and community indigeneity 17
Fig. S13. Correlations between ILK and land cover among communities by respondent gender and community indigeneity 18
Fig. S14. Estimated densities of ILK indices and land cover measures by cooperative forest clearing 19
Fig. S15. Proportion of presence of species across communities by household resource use and cooperative forest clearing 20
Fig. S16. Correlations between ILK and land cover among communities by kin networks 21
Fig. S17. Robustness to buffer size for land cover measures: Correlations between ILK and land cover by respondent gender and household resource use 22
Fig. S18. Robustness to household sample size: Correlations between ILK and land cover by respondent gender and household resource use 23
Fig. S19. Robustness to covariance within basins: Estimates of multi-level model by respondent gender and household resource use 24
Fig. S20. Robustness to covariance within basins and household sample size: Estimates of multi-level model by respondent gender and household resource use 25
Fig. S21. Nonparametric relationship of ILK with land cover 26
Fig. S22. Nonparametric relationship of ILK with land cover by respondent gender 27
Fig. S23. Nonparametric relationship of ILK with land cover by household resource use 28
Fig. S24. Correlations between ILK and land cover among communities by household resource use and community public work 29

Table S1. Indicator species 30
Table S2. Definition and characteristics of age, place-of-origin, leadership, and intensity/specialization analysis samples 31
Table S3. P-values for t test, Kolmogorov-Smirnov test, and correlations 32
Table S4. P-values for t test and correlations by cooperative forest clearing 33

Supplementary information text

S.1. Robustness check

The correlation results reported in Fig. 3 are robust to the size of the buffer (2km and 10km) used to construct land cover measures, with the exception of timber for 2km buffer in the resource use analysis sample (Fig. S17).

The different number of households sampled per community across respondent groups in the analysis sample (Table 1 and Table S2) may affect the concordance of ILK and land cover. In particular, a larger household sample size per community may enhance the concordance of collective knowledge at the community level. We repeated the construction of collective ILK measures, the concordance analysis, and the assessment of PCK focusing on two households (the first two according to household IDs) in each community for each respondent group. For each analysis sample, this alternative design mimics different targeting of two randomly selected respondents according to certain attributes. The results reported in Fig. S18 are similar to the original results in Fig. 3. As an exception, although the concordance for the game index (and a few game species) was weaker among resource users than nonusers in the original analysis, this pattern vanished. This is mainly because the correlations for nonusers, the sample size of which was greater than users, decreased. This indicates that the original results for game might reflect the concordance of collective knowledge affected by the distinct sample sizes of nonusers and users. This can also explain the similar patterns for game between main users and others (Fig. S9). Hence, distinct from timber and fish, PCK for game is considered high between users and nonusers.

The communities in the original and analysis samples are clustered by basins (Figs. 1 and S5). We ran a multi-level mixed-effects regression of ILK on land cover with a random intercept at the basin level, allowing covariance within basins. The results reported in Fig. S19 are similar to the original results in Fig. 3; as exceptions, the concordance for the game index (and some game species) was weaker among resource users than nonusers and the concordance for the timber index is similar between resource users and nonusers. When we repeated the analysis focusing on two households in each community for each respondent group as done for the correlation analysis above, the results reported in Fig. S20 are similar to the original results in Fig. 3 as well as Fig. S18. This is mainly because the point estimates for nonusers, the sample size of which was greater than users, decreased. These results indicate that the distinct patterns found for game and timber in the original multi-level model estimates (Fig. S19) could reflect the concordance of collective knowledge affected by the distinct sample sizes of nonusers and users. The results for fish are stable across the analyses. Overall, these results lend support to the main findings of the original correlation analysis (Fig. 3).

Since correlation analysis may not capture nonlinear relationships, we conducted a locally weighted regression of ILK on land cover. The results in the original sample show linear relationships generally for most species, and more so for species for which the correlation is stronger, especially for game and timber (Fig. S21). Here, we need to acknowledge the skewed distributions of land cover measures when interpreting the estimated Lowess (locally weighted scatterplot smoothing) smoothers (Fig. S4D, E). The nonparametric relationships are strikingly similar across three gender-related respondent groups, especially for game and timber (Fig. S22). In contrast, the nonparametric relationships are considerably different for resource users and nonusers for some species, notably for fish (Fig. S23). In particular, the estimated Lowess

smoothers are mostly higher and their slopes for timber and fish are steeper among users relative to nonusers, which are consistent with the means and correlations reported in Figs. 2 and 3.

S.2. Indigeneity

We repeated the assessment of PCK among Indigenous and Mestizo communities separately for comparison. According to local leaders in our community survey, the original sample consists of 130 Indigenous communities and 105 Mestizo communities. The location of these two types of communities is distinct, with Indigenous communities generally located further from cities than Mestizo communities (Takasaki et al. 2022). Among 15 ethnic groups in Indigenous communities, Kichwa, Shipibo-Konibo, and Kukama-Kukamiria peoples are common (over 70%). The household survey collected information about household indigeneity (self-reported identity). Although household Indigenous identity is uniform in about a half of communities, it is mixed in the remaining half and its distribution (the proportion of Indigenous households in the community) does not always match the community-level indigeneity reported in our community survey, depending on household sampling (Fig. S1O). We therefore focused on communities whose indigeneity is corroborated by household indigeneity (uniform or near uniform distributions, using 0.2 and 0.8 as cutoffs) – a total of 194 communities (102 Indigenous, 92 Mestizo). The choice of cutoffs is based on the size of each respondent group in the remaining analysis samples. We repeated the assessment of PCK among these Indigenous and Mestizo communities separately. This heterogeneity analysis is infeasible for the resource use analysis sample due to the small sample size (Table 1).

S.3. Cooperative labor exchange

We considered community public works (e.g., building of a community house, street maintenance) as an alternative cooperative labor institution which is organized at the community level rather than along kinship lines, and in which people do not generally engage with nature or share food. We categorized communities into high and low intensity types according to the number of community public works undertaken per month using median (4) as a cutoff (this information, which was collected in another community survey associated with the household survey, is missing in 2 communities). The assessment of PCK according to resource use is not differentiated by the intensity of community public work (Fig. S24). As an exception, the stronger concordance among resource users than nonusers for fish is observed only in communities with high intensity of community public work and the concordance among nonusers is not statistically significant there; in contrast, the concordance for fish is statistically significant for both users and nonusers in low intensity communities. These patterns are rather opposite to what is found for cooperative forest clearing.

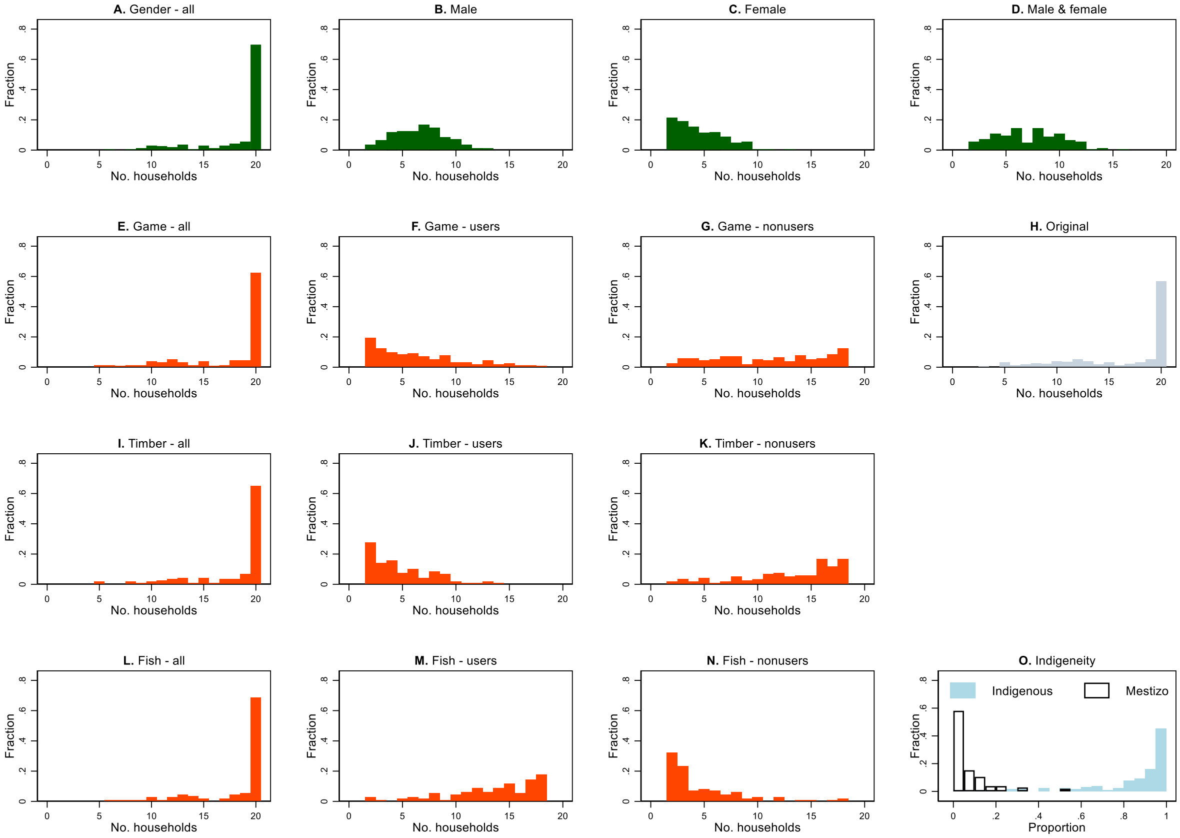

Fig. S1. Histogram plots of household sample size and indigeneity. (**A-N**) Number of households per community covered in household survey among communities; (**O**) Proportion of Indigenous households in communities by community indigeneity. The samples are all (**A**), male (**B**), female (**C**), and male-and-female (**D**) respondents in the gender analysis sample; all (**E**), resource users (**F**), and nonusers (**G**) in the resource use analysis sample for game; all (**I**), resource users (**J**), and nonusers (**K**) in the resource use analysis sample for timber; all (**L**), resource users (**M**), and nonusers (**N**) in the resource use analysis sample for fish; and the original sample of the household survey (**H, O**). See Table S1 for the definitions of the analysis samples and respondent groups, and their summary statistics.

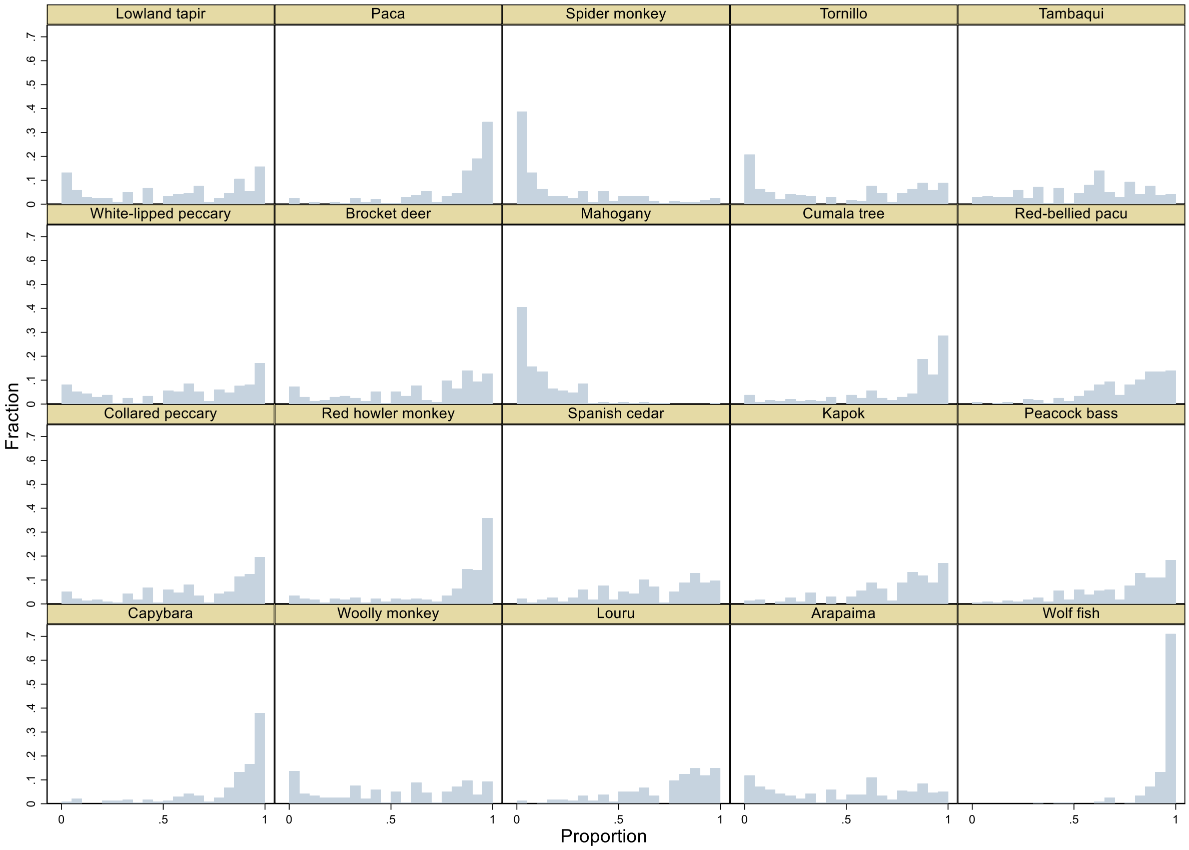

Fig. S2. Histogram plots of proportion of presence of species across communities. The proportion of households in the community reporting the presence of each of 20 indicator species (i.e., ILK) across communities are shown. The sample is the original sample of the household survey.

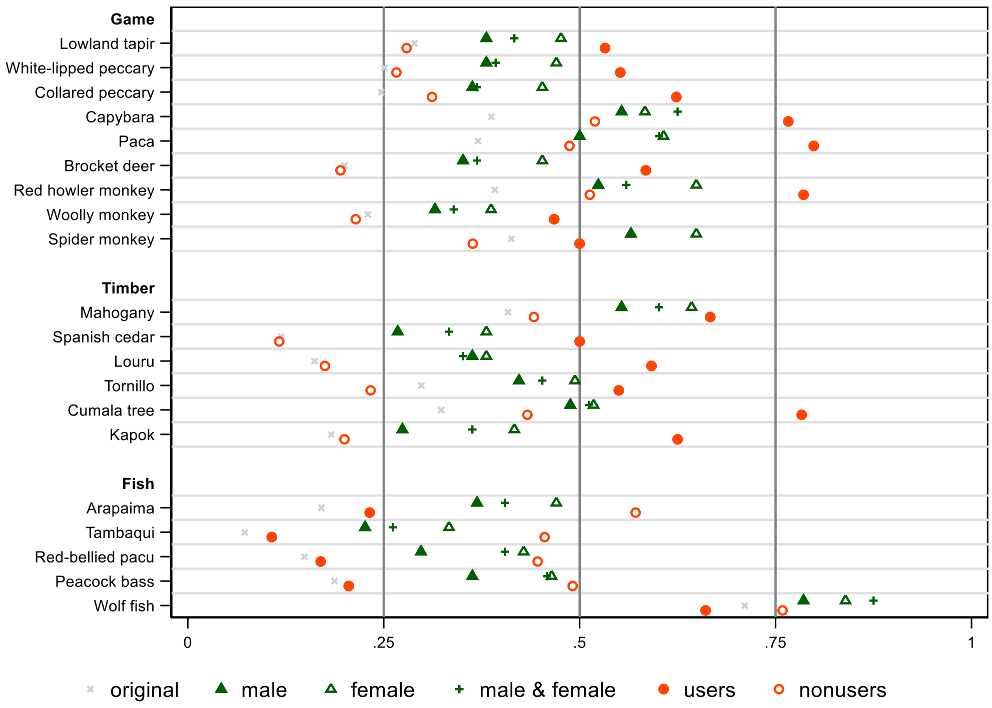

Fig. S3. Proportion of communities where ILK among respondents was uniform by respondent gender and household resource use. The proportion of communities where households unanimously reported the presence or absence of each of 20 indicator species in the community is shown for each sample. Colors represent different samples. The samples are the original sample of the household survey; male, female, and male-and-female respondents in the gender analysis sample; and resource users and nonusers in the resource use analysis sample for game, timber, and fish. See Table 1 for the definitions of the analysis samples and respondent groups, and their summary statistics.

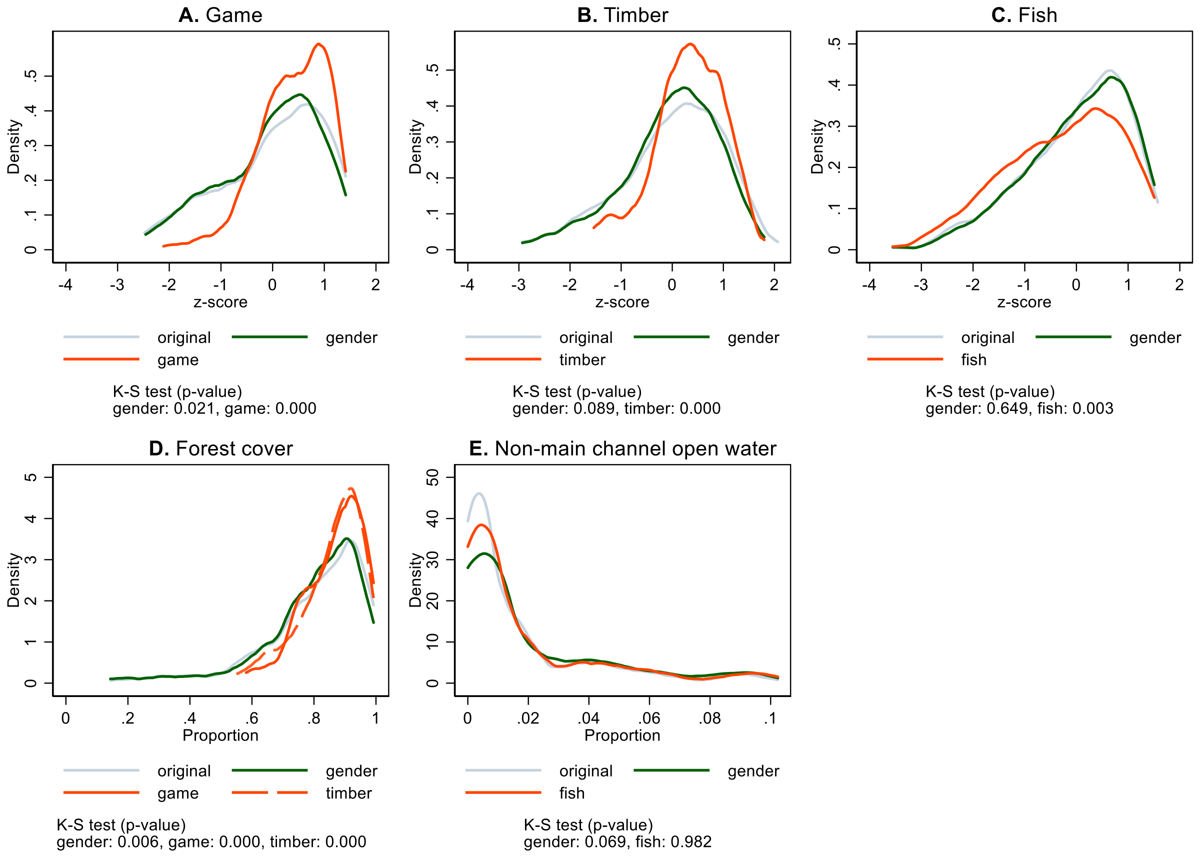

Fig. S4. Estimated densities of ILK indices and land cover measures across samples. (**A**) Game index; (**B**) Timber index; (**C**) Fish index; (**D**) Forest cover; and (**E**) Non-main channel open water. See the caption to Fig. 3 for ILK indices and land cover measures. The samples are original sample (**A-E**), gender analysis sample (**A-E**), resource use analysis sample for game (**A, D**), timber (**B, D**), and fish (**C, E**). P-values for Kolmogorov-Smirnov (K-S) tests for the equality of distributions between each analysis sample and the remaining sample in the original sample of the household survey are reported. For example, 'gender' compares gender analysis sample and the remaining sample in the original sample. See Table 1 for the definitions of the analysis samples and respondent groups, and their summary statistics.

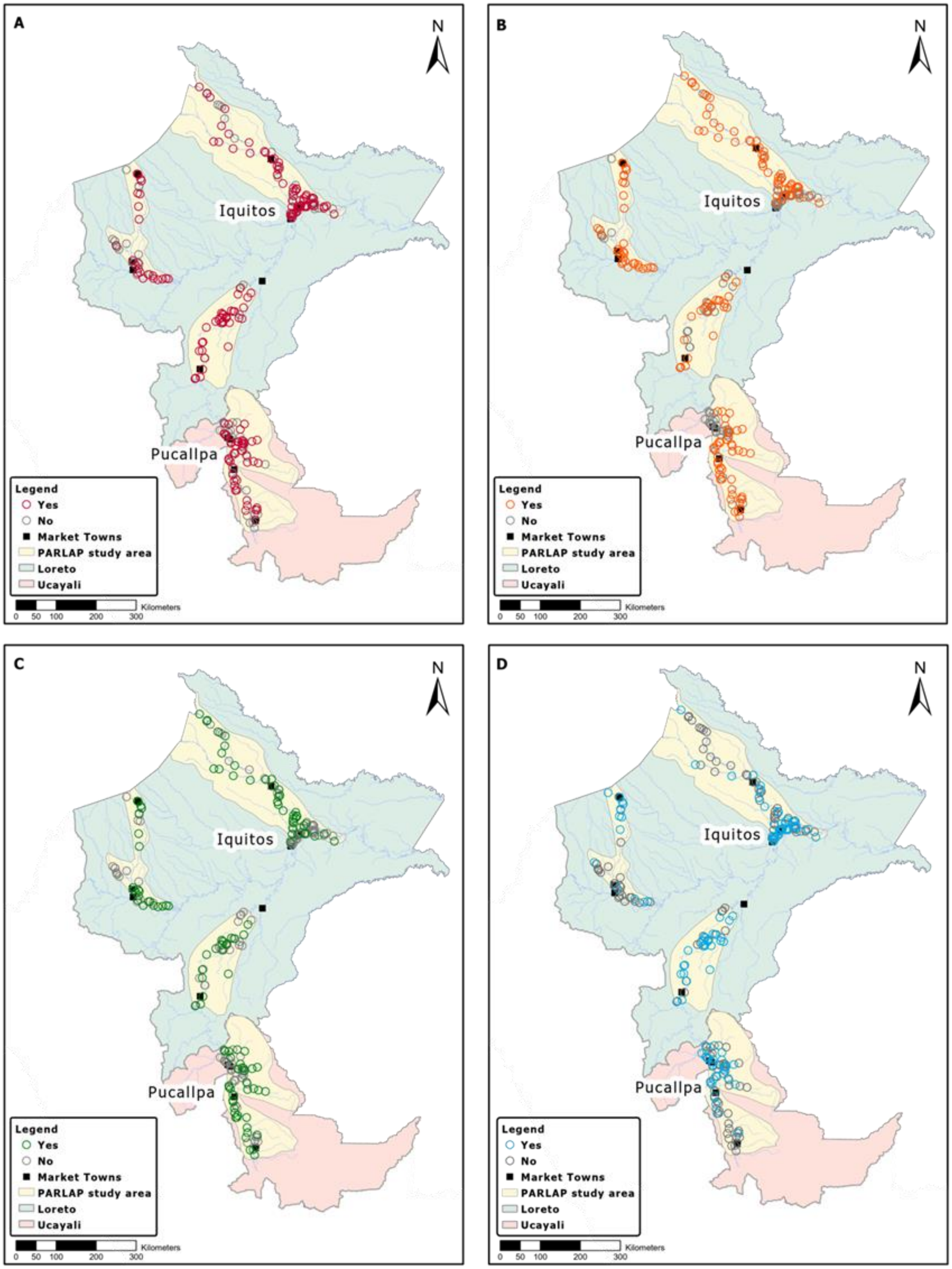

Fig. S5. Communities included in the analysis samples. (**A**) Gender analysis sample; Resource use analyis sample for (**B**) game, (**C**) timber, and (**D**) fish. See the caption to Fig. 1 for general information. See Table 1 for the definitions of the analysis samples and respondent groups, and their summary statistics.

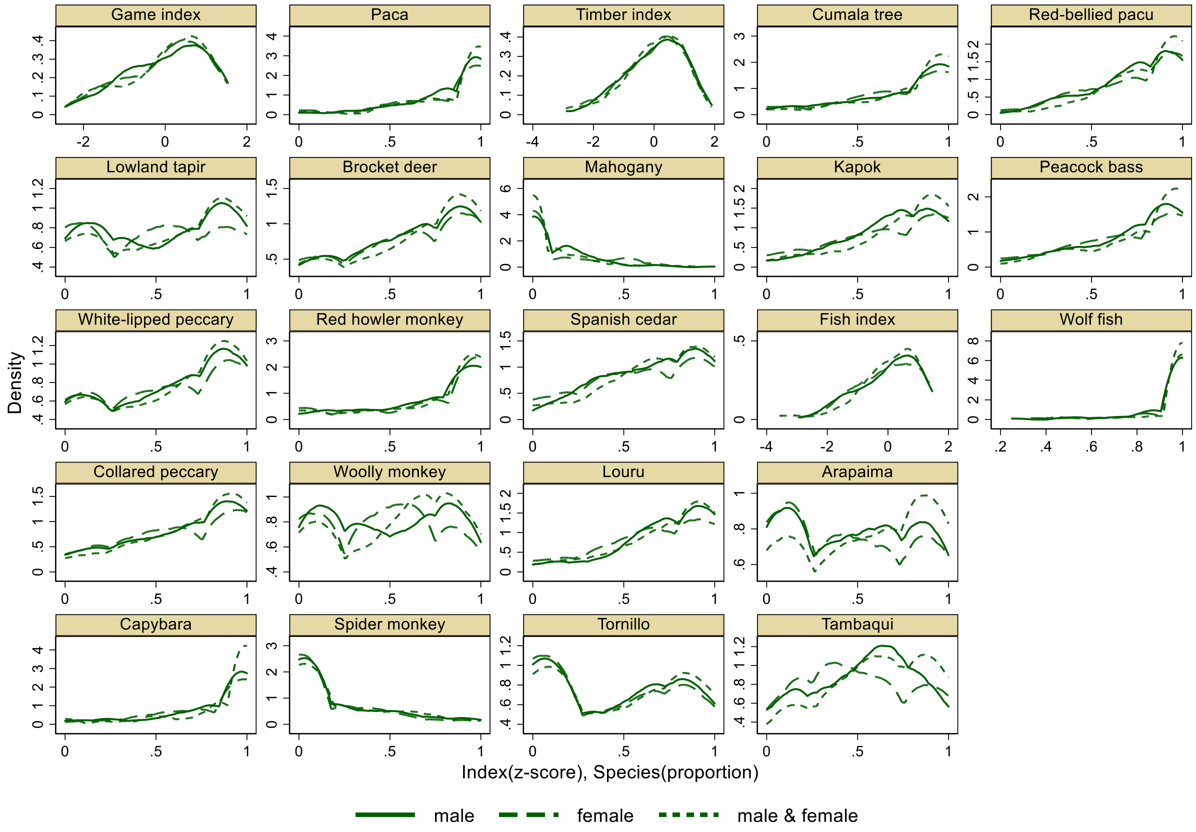

Fig. S6. Estimated densities of ILK measures by respondent gender. See the caption to Fig. 3 for ILK and land cover measures. The samples are male, female, and male-and-female respondents in the gender analysis sample. See Table 1 for the definitions of the analysis sample and respondent groups, and their summary statistics.

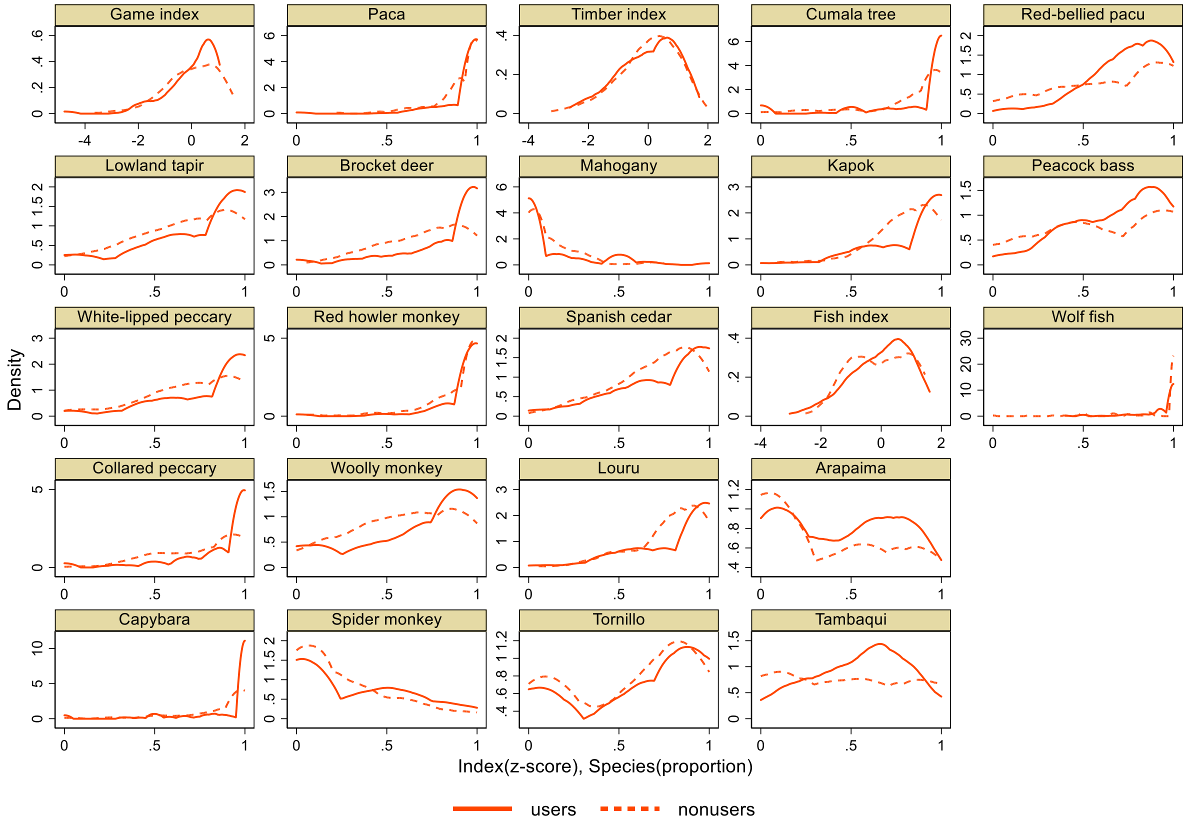

Fig. S7. Estimated densities of ILK measures by household resource use. See the caption to Fig. 3 for ILK and land cover measures. The samples are resource users and nonusers in the resource use analysis samples for game, timber, and fish. See Table 1 for the definitions of the analysis samples and respondent groups, and their summary statistics.

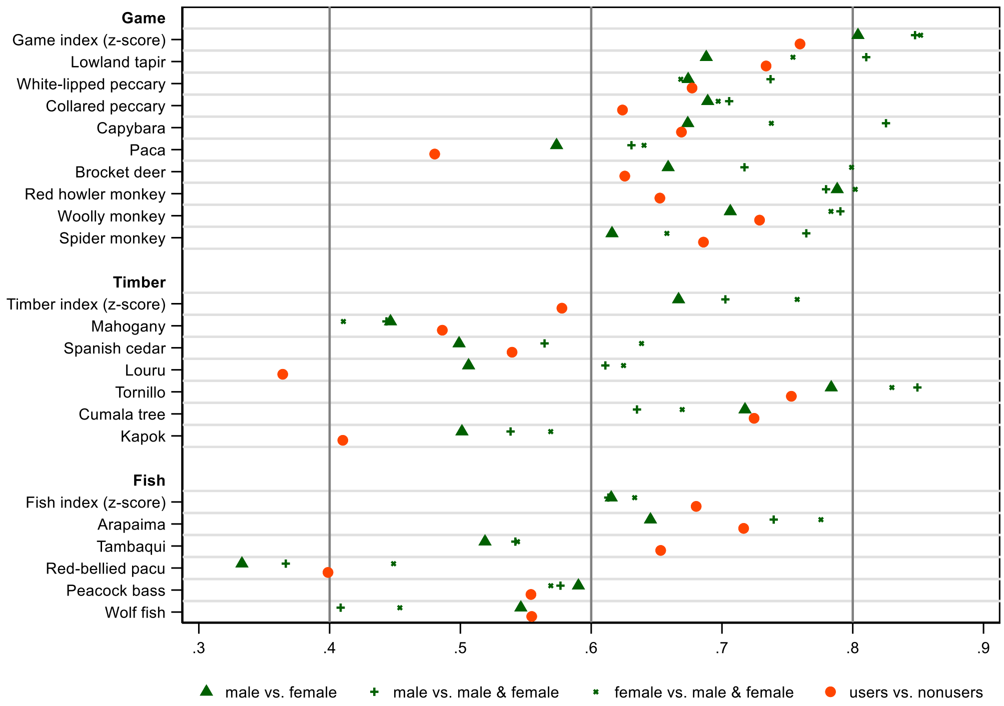

Fig. S8. Correlations of ILK measures among communities across respondent gender and household resource use. See the caption to Fig. 3 for ILK measures. Pearson's correlation coefficients of ILK measures between respondent groups among communities are shown. Colors represent different samples. The samples are male, female, and male-and-female respondents in the gender analyis sample; and resource users and nonusers in the resource use analysis samples for game, timber, and fish. See Table 1 for the definitions of the analysis samples and respondent groups, and their summary statistics.

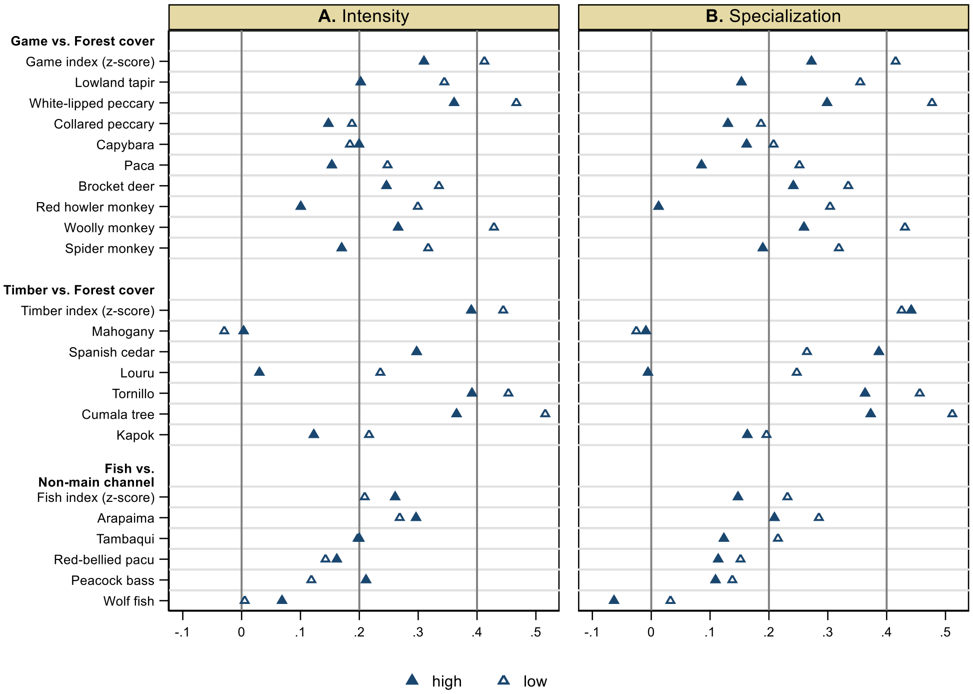

Fig. S9. Correlation between ILK and land cover among communities by household harvest intensity and specialization. See the caption to Fig. 3 for ILK and land cover measures. Pearson's correlation coefficients between the ILK measures and land cover measures among communities are shown for each sample. The samples are households with high and low harvest intensity (**A**) and specialization (**B**) in the intensity/specialization analysis samples for game, timber, and fish. See Table S2 for the definitions of the analysis samples and respondent groups, and their summary statistics.

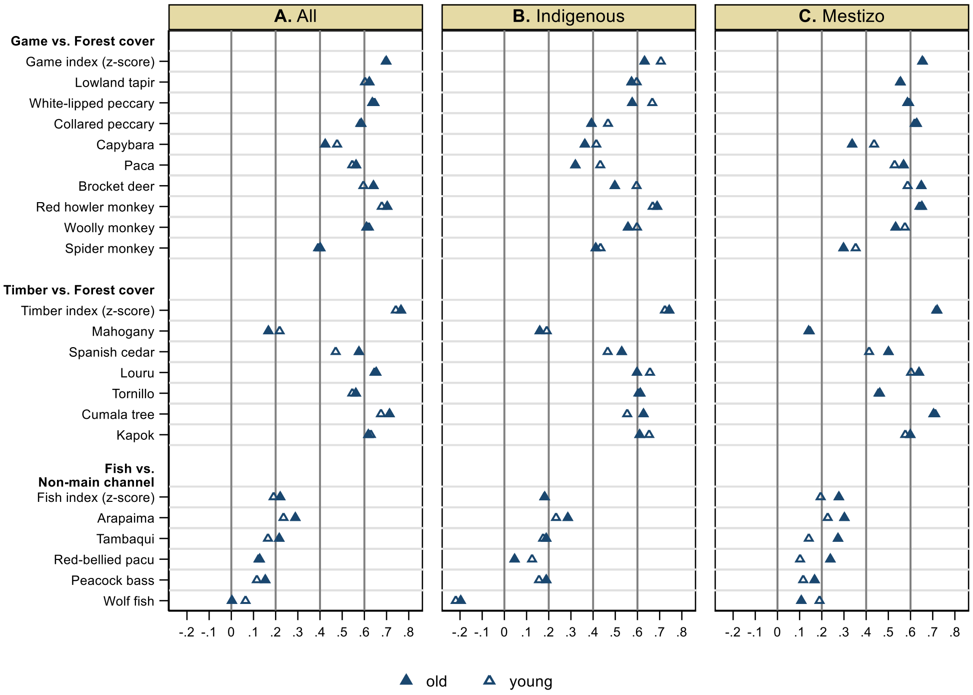

Fig. S10. Correlations between ILK and land cover among communities by respondent age and community indigeneity. See the caption to Fig. 3 for ILK and land cover measures. Pearson's correlation coefficients between the ILK measures and land cover measures among communities are shown for each sample. The samples are old and young respondents (median age, 42, as a cutoff) in the age analyis sample in all (**A**), Indigenous (**B**), and Mestizo (**C**) communities. See Table S2 for the definitions of the analysis sample and respondent groups, and their summary statistics for all communities. See S.2. Indigeneity for the definitions of Indigenous and Mestizo communities.

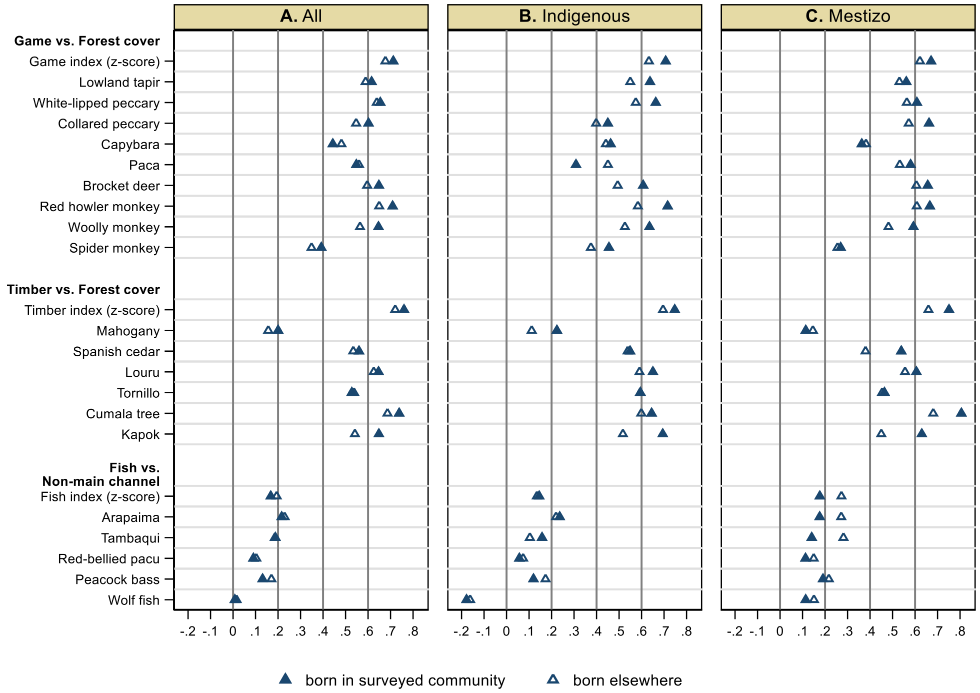

Fig. S11. Correlation between ILK and land cover among communities by respondent place of origin and community indigeneity. See the caption to Fig. 3 for ILK and land cover measures. Pearson's correlation coefficients between the ILK measures and land cover measures among communities are shown for each sample. The samples are respondents who were born in the surveyed community and elsewhere in the place-of-origin analyis sample in all (**A**), Indigenous (**B**), and Mestizo (**C**) communities. See Table S2 for the definitions of the analysis sample and respondent groups, and their summary statistics for all communities. See S.2. Indigeneity for the definitions of Indigenous and Mestizo communities.

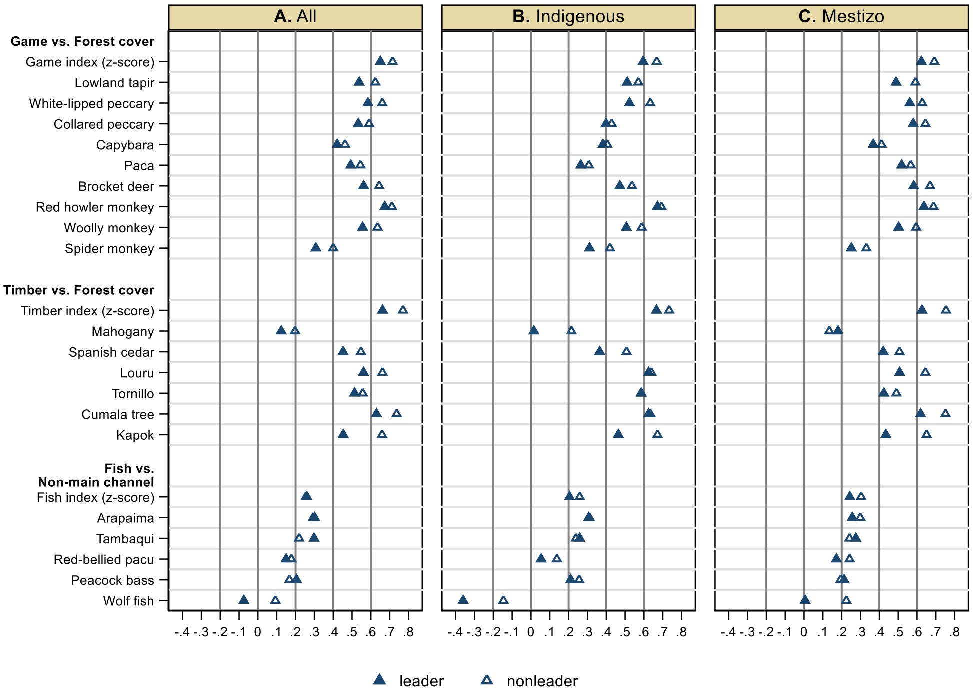

Fig. S12. Correlations between ILK and land cover among communities by household head leadership in the community and community indigeneity. See the caption to Fig. 3 for ILK and land cover measures. Pearson's correlation coefficients between the ILK measures and land cover measures among communities are shown for each sample. The sample is leader and nonleader households in the leadership analysis sample in all (**A**), Indigenous (**B**), and Mestizo (**C**) communities. See Table S2 for the definitions of the analysis sample and respondent groups, and their summary statistics for all communities. See S.2. Indigeneity for the definitions of Indigenous and Mestizo communities.

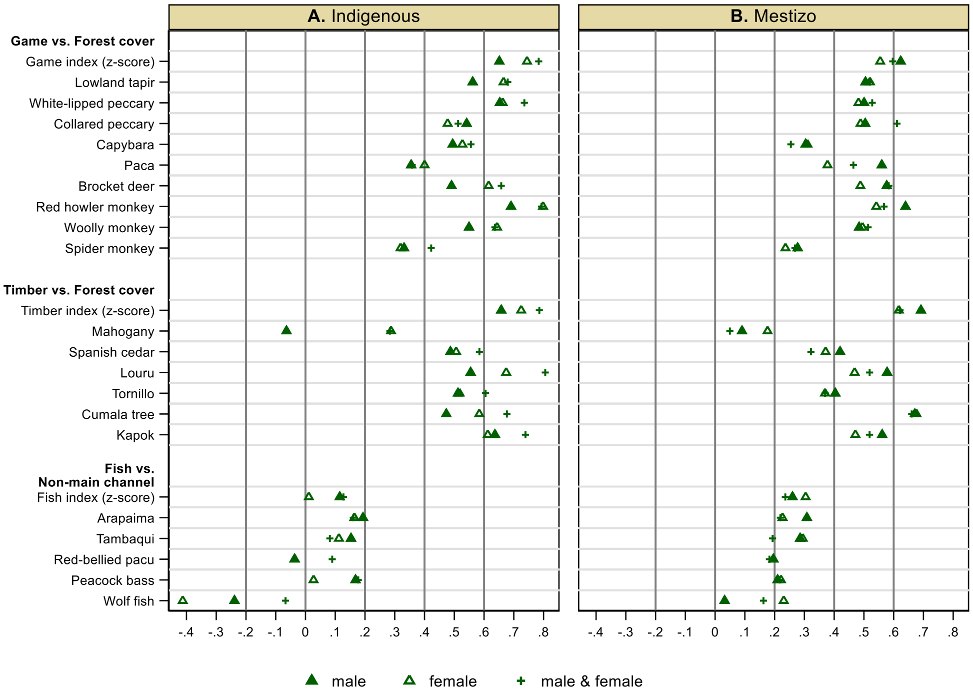

Fig. S13. Correlations between ILK and land cover among communities by respondent gender and community indigeneity. See the caption to Fig. 3 for ILK and land cover measures. Pearson's correlation coefficients between the ILK measures and land cover measures among communities are shown for each sample. The samples are male, female, and male-and-female respondents in the gender analysis sample in Indigenous (**A**) and Mestizo (**B**) communities. See Table 1 for the definitions of the analysis sample and respondent groups. See S.2. Indigeneity for the definitions of Indigenous and Mestizo communities.

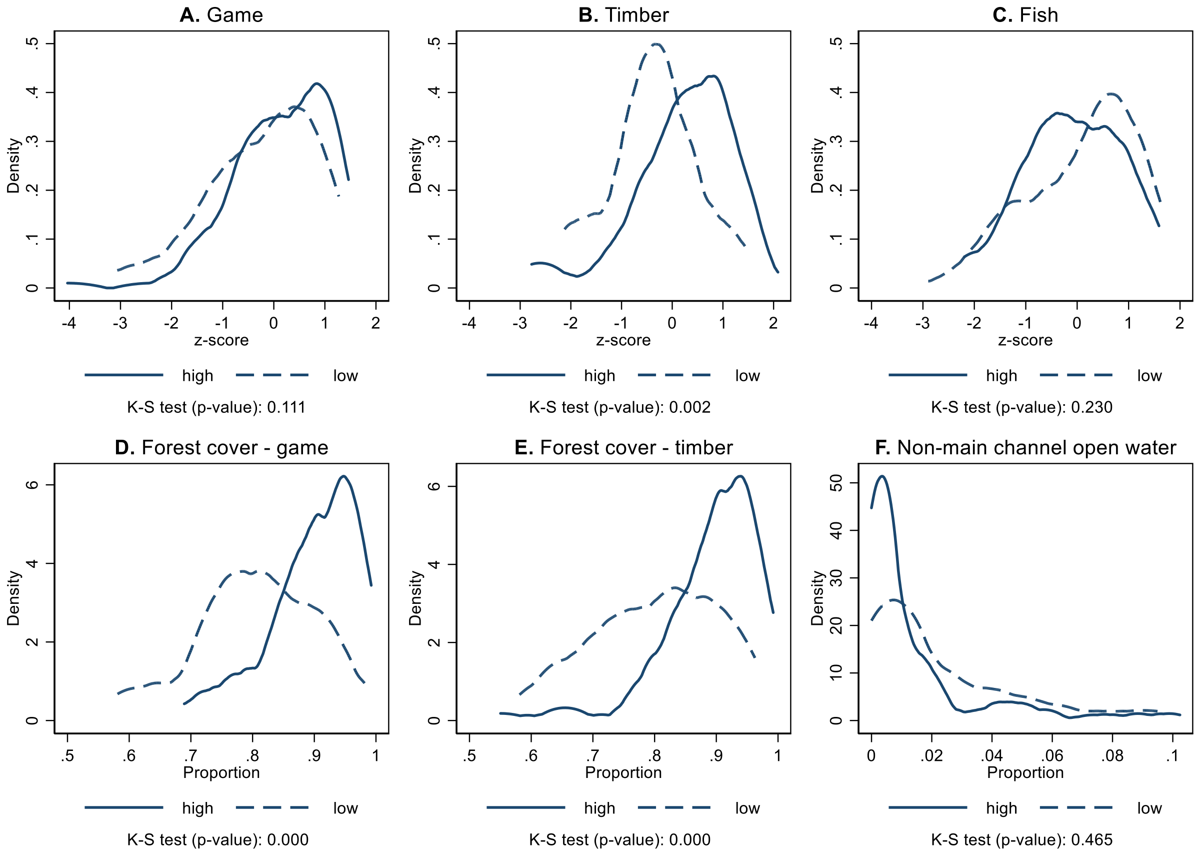

Fig. S14. Estimated densities of ILK indices and land cover measures by cooperative forest clearing. (**A**) Game index; (**B**) Timber index; (**C**) Fish index; (**D, E**) Forest cover; and (**F**) Non-main channel open water. See the caption to Fig. 3 for ILK indices and land cover measures. The samples are the resource use analysis sample for game (**A, D**), timber (**B, E**), and fish (**C, F**) in communities with low and high intensity of cooperative forest clearing. See Table 1 for the definitions of the analysis samples and respondent groups, and their summary statistics. See the caption to Fig. 4 for the definitions of high and low intensity communities. P-values for Kolmogorov-Smirnov (K-S) tests for the equality of distributions between communities with low and high intensity of cooperative forest clearing are reported.

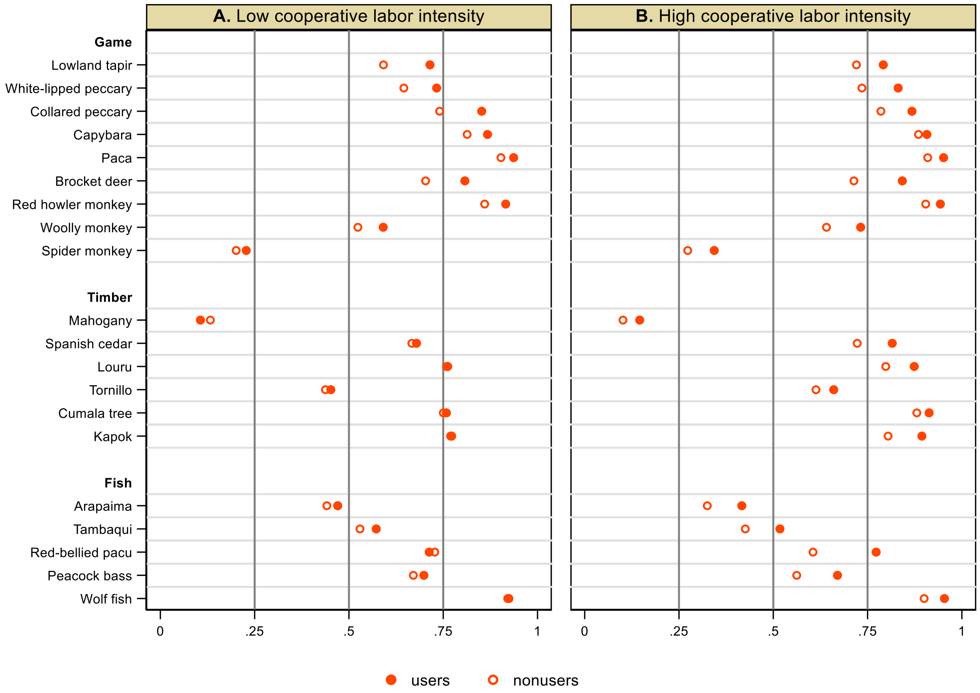

Fig. S15. Proportion of presence of species across communities by household resource use and cooperative forest clearing. The means of the proportion of households in the community reporting the presence of each of 20 indicator species (i.e., ILK) across communities are shown for each sample. The samples are resource users and nonusers in the resource use analyis samples for game, timber, and fish in communities with low (**A**) and high (**B**) intensity of cooperative forest clearing. See Table 1 for the definitions of the analysis samples and respondent groups, and their summary statistics. See the caption to Fig. 4 for the definitions of high and low intensity communities.

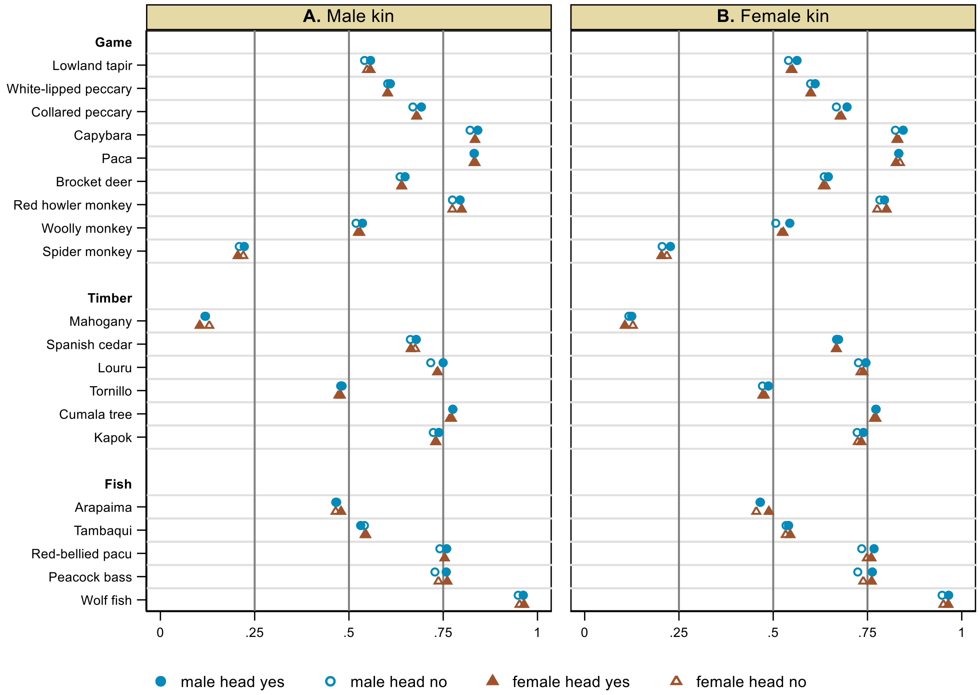

Fig. S16. Proportion of presence of species across communities by kin networks. The means of the proportion of households in the community reporting the presence of each of 20 indicator species (i.e., ILK) across communities are shown for each sample. The samples are households with male head with male kin (yes), male head without male kin (no), female head with male kin (yes), and female head without male kin (no) (**A**); households with male head with female kin (yes), male head without female kin (no), female head with female kin (yes), and female head without female kin (no) (**B**) in the kin network analysis sample. See Table 1 for the definitions of the analysis sample and respondent groups, and their summary statistics.

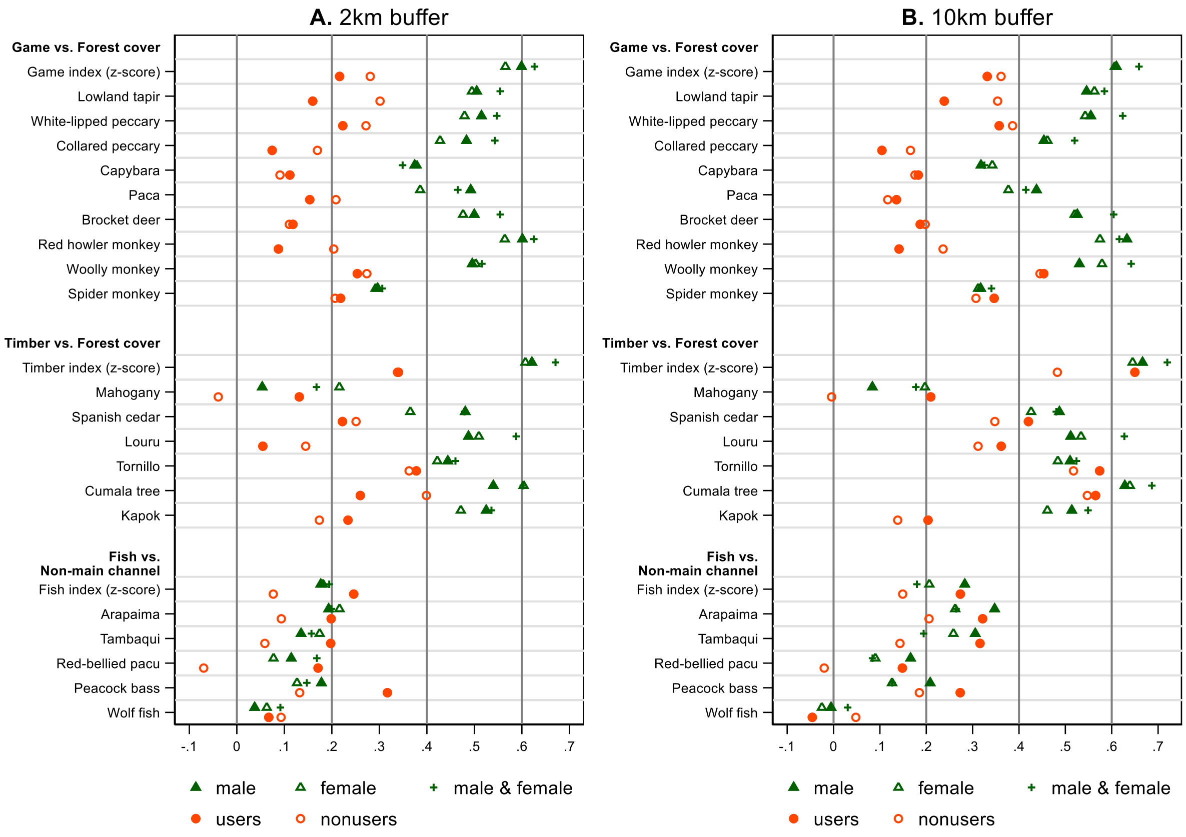

Fig. S17. Robustness to buffer size for land cover measures: Correlations between ILK and land cover by respondent gender and household resource use. (**A**) 2km buffer; (**B**) 10km buffer. See the caption to Fig. 3 for ILK measures. Forest cover and non-main channel open water, respectively, are measured by the proportion of forest on land and non-main channel open water in a 2 km buffer and a 10 km buffer centered on each community. Pearson's correlation coefficients between the ILK measures and land cover measures among communities are shown for each sample. Colors represent different samples. The samples are male, female, and male-and-female respondents in the gender analysis sample; and resource users and nonusers in the resource use analysis sample for game, timber, and fish. See Table 1 for the definitions of the analysis samples and respondent groups, and their summary statistics.

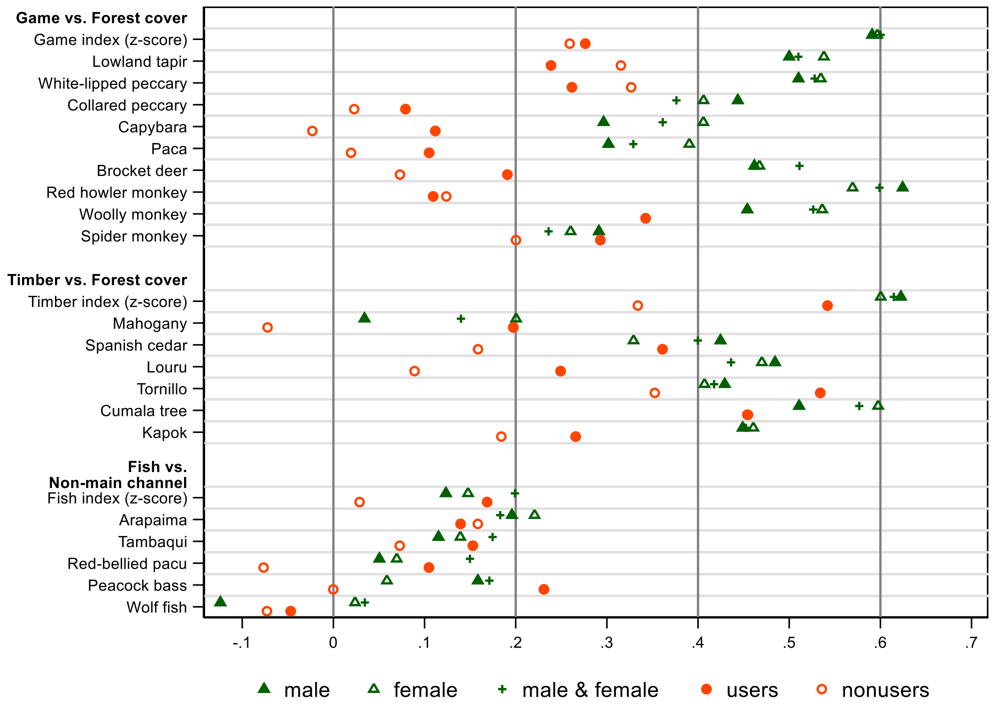

Fig. S18. Robustness to household sample size: Correlations between ILK and land cover by respondent gender and household resource use. See the caption to Fig. 3 for ILK and land cover measures. Pearson's correlation coefficients between the ILK measures and land cover measures among communities are shown for each sample. Colors represent different samples. The samples are two male, two female, and two male-and-female respondents in the gender analysis sample; and two resource users and two nonusers in the resource use analysis sample for game, timber, and fish. See Table 1 for the definitions of the analysis samples and respondent groups, and their summary statistics (two households per community).

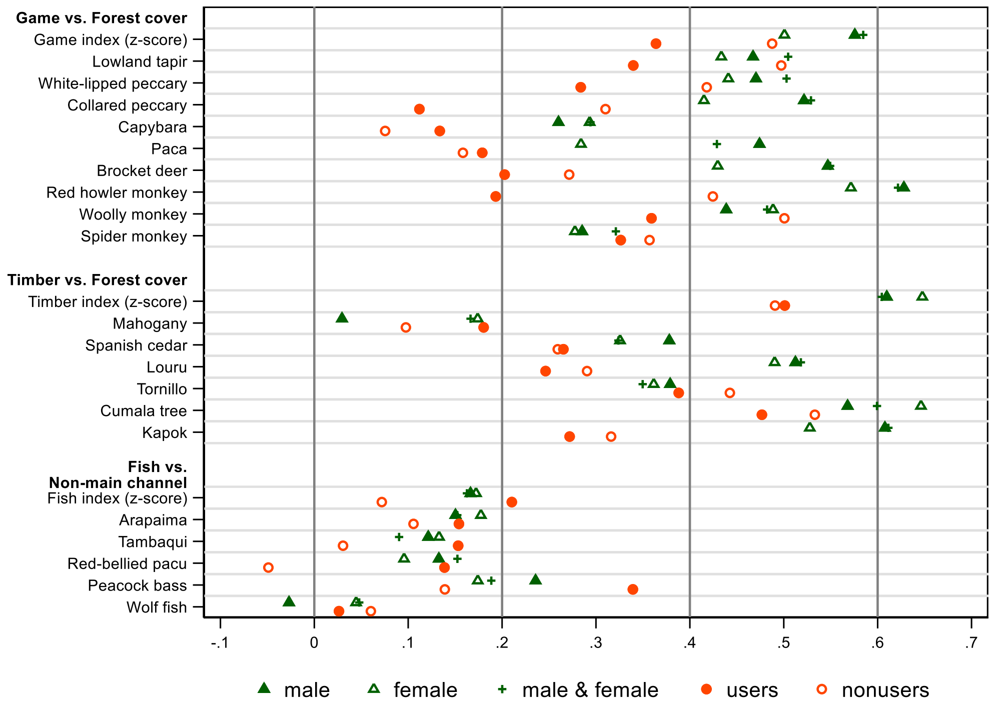

Fig. S19. Robustness to covariance within basins: Estimates of multi-level model by respondent gender and household resource use. See the caption to Fig. 3 for ILK and land cover measures. The estimated standardized coefficients for land cover from a multi-level mixed-effects regression of ILK measures with a random intercept at the basin level is shown for each sample. Colors represent different samples. The samples are male, female, and male-and-female respondents in the gender analysis sample; and resource users and nonusers in the resource use analysis samples for game, timber, and fish. See Table 1 for the definitions of the analysis samples and respondent groups, and their summary statistics.

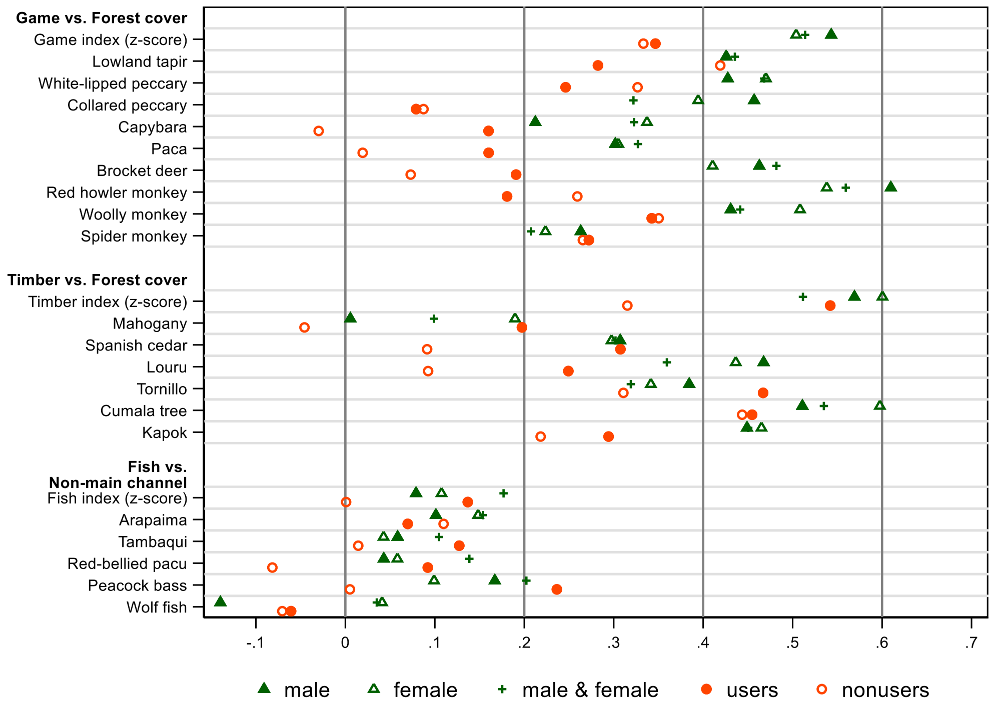

Fig. S20. Robustness to covariance within basins and household sample size: Estimates of multi-level model by respondent gender and household resource use. See the caption to Fig. 3 for ILK and land cover measures. The estimated standardized coefficients for land cover from a multi-level mixed-effects regression of ILK measures with a random intercept at the basin level are shown for each sample. Colors represent different samples. The samples are two male, two female, and two male-and-female respondents in the gender analysis sample; and two resource users and two nonusers in the resource use analysis sample for game, timber, and fish. See Table 1 for the definitions of the analysis samples and respondent groups, and their summary statistics (two households per community).

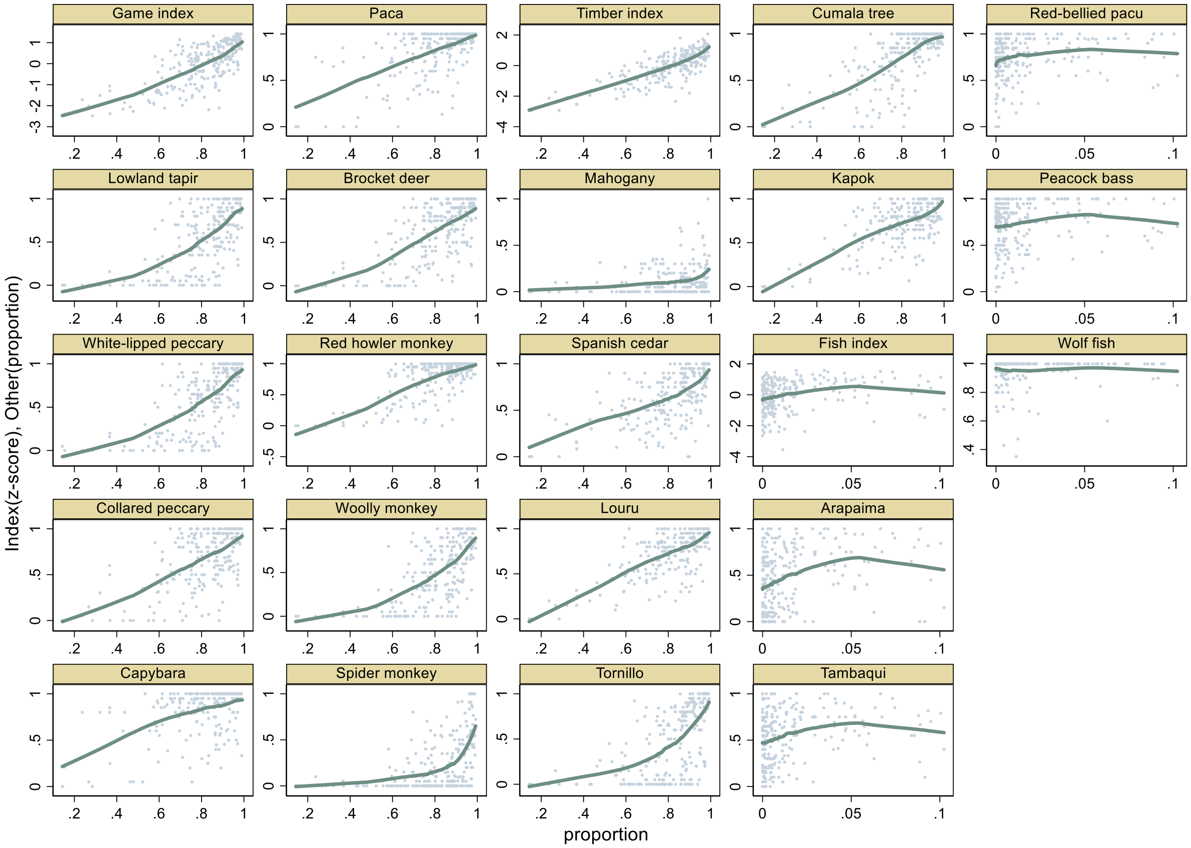

Fig. S21. Nonparametric relationship of ILK with land cover. See the caption to Fig. 3 for ILK and land cover measures. Lowess (locally weighted scatterplot smoothing) smoothers are shown for each ILK measure. ILK is on the y-axis and corresponding land cover is on the x-axis. Points are perturbated for exposition. The sample is the original sample of the household survey.

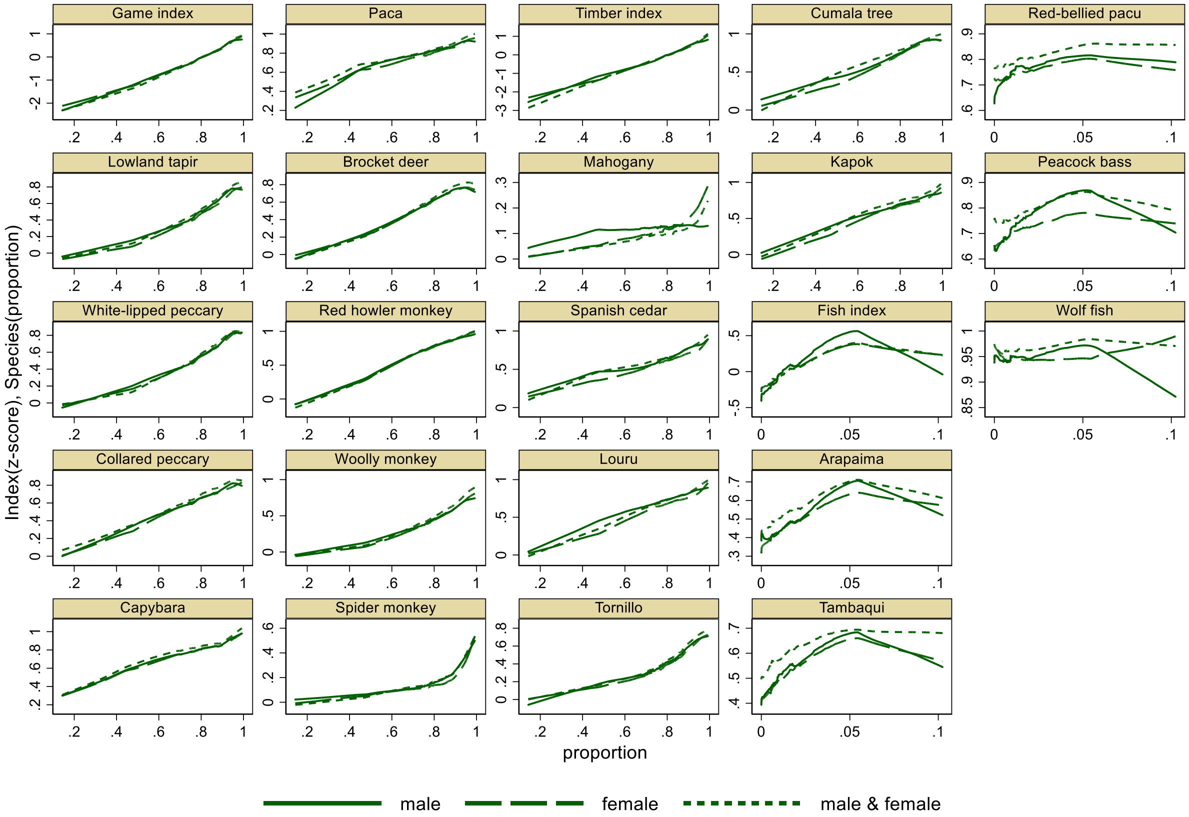

Fig. S22. Nonparametric relationship of ILK with land cover by respondent gender. See the caption to Fig. 3 for ILK and land cover measures. Lowess (locally weighted scatterplot smoothing) smoothers are shown for each ILK measure. ILK is on the y-axis and corresponding land cover is on the x-axis. The samples are male, female, and male-and-female respondents in the gender analysis sample. See Table 1 for the definitions of the analysis sample and respondent groups, and their summary statistics.

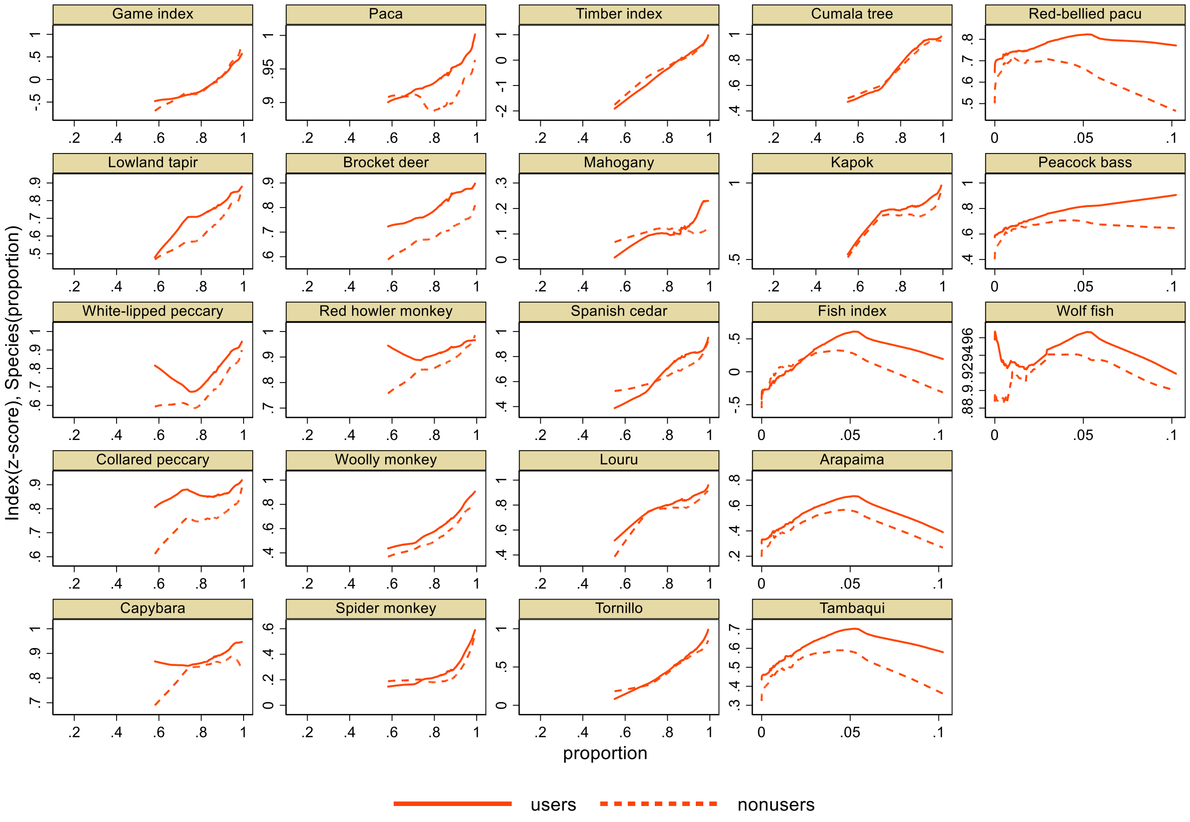

Fig. S23. Nonparametric relationship of ILK with land cover by household resource use. See the caption to Fig. 3 for ILK and land cover measures. Lowess (locally weighted scatterplot smoothing) smoothers are shown for each ILK measure. ILK is on the y-axis and corresponding land cover is on the x-axis. The samples are resource users and nonusers in the resource use analysis samples for game, timber, and fish. See Table 1 for the definitions of the analysis samples and respondent groups, and their summary statistics.

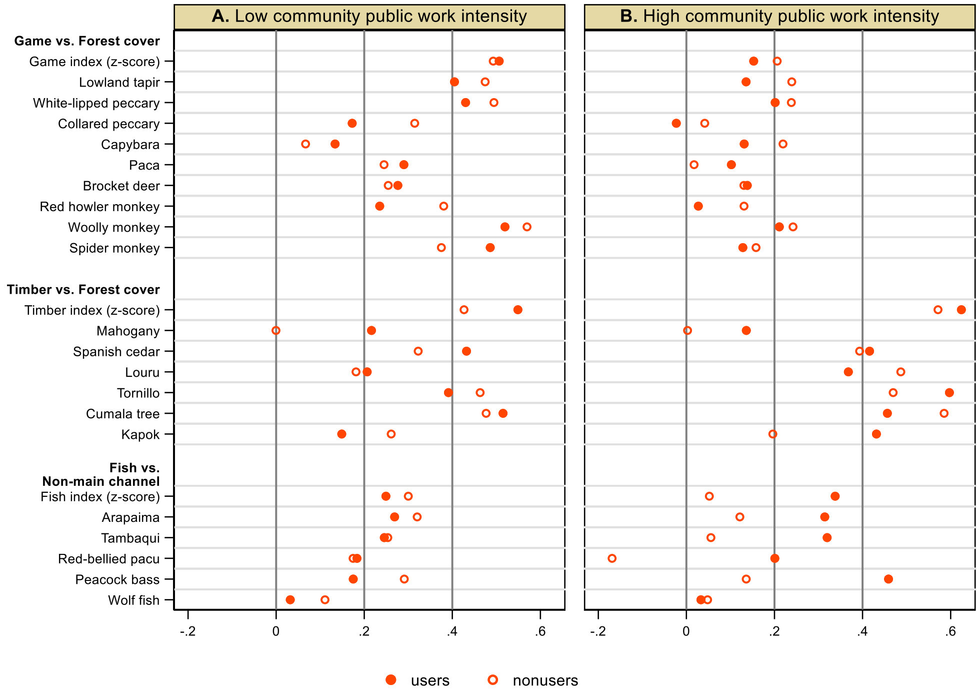

Fig. S24. Correlations between ILK and land cover among communities by household resource use and community public work. See the caption to Fig. 3 for ILK and land cover measures. Pearson's correlation coefficients between the ILK measures and land cover measures among communities are shown for each sample. The samples are resource users and nonusers in the resource use analyis samples for game, timber, and fish in communities with low (**A**) and high (**B**) intensity of community public work. See Table 1 for the definitions of the analysis samples and respondent groups. High and low intensity communities undertook a large and small number of community public works per week (median number, 4, as a cutoff) (see S.3. Cooperative labor exchange for details).

Table S1. Indicator species

	English common name	Spanish common name	Scientific name	Species classification
Game:				
	Lowland tapir	Sacha vaca	*Tapirus terrestris*	Large-bodied
	White-lipped peccary	Huangana	*Tayassu pecari*	Large-bodied
	Collared peccary	Sajino	*Pecari tajacu*	Large-bodied
	Capybara	Ronsoco	*Hydrochoerus hydrochaeris*	Large-bodied
	Paca	Majás	*Cuniculus paca*	Small-bodied
	Brocket deer	Venado	*Mazama spp.*	Large-bodied
	Red howler monkey	Mono coto	*Alouatta seniculus*	Monkey
	Woolly monkey	Mono choro	*Lagothrix lagotricha*	Monkey
	Spider monkey	Mono maquisapa	*Ateles paniscus*	Monkey
Timber:				
	Mahogany	Caoba	*Swietenia macrophylla*	1st class
	Spanish cedar	Cedro	*Cedrela odorata*	1st class
	Louru	Moena	*Ocotea bofo*	1st class
	Tornillo	Tornillo	*Cedrelinga cateniformis*	1st class
	Cumala tree	Cumala	*Osteophloe platyspermum*	2nd class
	Kapok	Lupuna	*Ceiba pentandra*	2nd class
Fish:				
	Arapaima	Paiche	*Arapaima gigas*	1st class
	Tambaqui	Gamitana	*Colossoma macropomum*	1st class
	Red-bellied pacu	Paco	*Piaractus brachypomus*	2nd class
	Peacock bass	Tucunaré	*Cichla ocellaris*	2nd class
	Wolf fish	Fasaco	*Hoplias malabaricus*	2nd class

Table S2. Definition and characteristics of age, place-of-origin, leadership, and intensity/specialization analysis samples

	Definition of analysis samples	Summary statistics						
		Original sample		Analysis sample				
		Proportion	No. Households	Proportion	No. Households	No. Communities	Mean no. households per community	
	(1)	(2)	(3)	(4)	(5)	(6)	(7)	(8)
Respondent age	Old vs. young, using median age (42) as a cutoff (mean age is used for households with two respondents)	Old<br>0.51	3924	Old<br>0.51	3872	227	Old<br>8.7	Young<br>8.4
Respondent place of origin	Surveyed community vs. elsewhere, depending on whether at least one respondent was born in the surveyed community	Yes<br>0.53	3924	Yes<br>0.55	3517	201	Yes<br>9.7	No<br>7.8
Household leadership	Leader vs. nonleader, depending on whether the head of household or spouse has ever held a primary leadership position	Leader<br>0.31	3922	Leader<br>0.32	3707	213	Leader<br>5.6	Nonleader<br>11.8
Household harvest intensity/specialization				High			High	Low
Game	High (top 2 users) and low (others, including both users and nonusers) according to income ranking (intensity) and income share ranking (specialization) in the community for hunting, timber harvesting, and fishing			0.12	1951	114	2.0	15.1
Timber				0.11	1295	72	2.0	16.0
Fish				0.12	3841	229	2.0	14.8

The proportion of each attribute across households (along with household sample size) is in column 2 for the original sample of the household survey and column 4 for corresponding analysis samples. Household sample size for some attributes is slightly smaller than 3929 due to missing values. The proportion of high resource use intensity/specialization is not available for the original sample because it is not defined in communities with no or limited households with high resource use intensity/specialization. For each analysis sample, community sample size is in column 6 and the mean number of households per community in each respondent type is in columns 7 and 8.

Table S3. P-values for t test, Kolmogorov-Smirnov test, and correlations

		t test in Fig. 2				K-S test in Figs. S6 and S7				Correlation in Fig. 3					
Analysis sample		Gender			Resource use	Gender			Resource use	Original	Gender			Resource use	
Respondent type		Male vs. female	Male vs. male & female	Female vs. male & female	Users vs. nonusers	Male vs. female	Male vs. male & female	Female vs. male & female	Users vs. nonusers		Male	Female	Male & female	Users	Nonusers
		(1)	(2)	(3)	(4)	(5)	(6)	(7)	(8)	(9)	(10)	(11)	(12)	(13)	(14)
Game:	Game index (z-score)	n.a.	n.a.	n.a.	n.a.	0.927	0.696	0.991	0.086	**0.000**	**0.000**	**0.000**	**0.000**	**0.000**	**0.000**
	Lowland tapir	0.146	0.150	**0.004**	**0.000**	0.290	0.927	0.112	**0.000**	**0.000**	**0.000**	**0.000**	**0.000**	**0.001**	**0.000**
	White-lipped peccary	0.513	0.457	0.195	**0.000**	0.927	0.927	0.431	**0.000**	**0.000**	**0.000**	**0.000**	**0.000**	**0.000**	**0.000**
	Collared peccary	0.705	**0.042**	**0.019**	**0.000**	0.515	0.927	0.233	**0.000**	**0.000**	**0.000**	**0.000**	**0.000**	0.370	**0.027**
	Capybara	0.758	**0.013**	**0.017**	**0.023**	0.785	0.356	0.112	**0.001**	**0.000**	**0.000**	**0.000**	**0.000**	0.094	0.125
	Paca	0.366	0.160	**0.024**	**0.001**	0.696	0.145	0.785	**0.000**	**0.000**	**0.000**	**0.000**	**0.000**	0.052	0.093
	Brocket deer	0.793	0.134	**0.040**	**0.000**	0.864	0.604	0.515	**0.000**	**0.000**	**0.000**	**0.000**	**0.000**	**0.012**	**0.016**
	Red howler monkey	0.722	0.586	0.365	**0.000**	0.696	0.785	0.785	**0.000**	**0.000**	**0.000**	**0.000**	**0.000**	0.126	**0.001**
	Woolly monkey	0.587	**0.020**	**0.004**	**0.000**	0.927	0.290	0.086	**0.003**	**0.000**	**0.000**	**0.000**	**0.000**	**0.000**	**0.000**
	Spider monkey	0.150	0.738	0.071	**0.007**	0.604	1.000	0.696	**0.035**	**0.000**	**0.000**	**0.000**	**0.000**	**0.000**	**0.001**
Timber:	Timber index (z-score)	n.a.	n.a.	n.a.	n.a.	0.969	0.969	0.785	0.888	**0.000**	**0.000**	**0.000**	**0.000**	**0.000**	**0.000**
	Mahogany	0.688	0.218	0.137	0.341	0.356	0.145	0.515	**0.011**	**0.000**	0.327	**0.005**	**0.019**	**0.049**	0.994
	Spanish cedar	0.085	0.440	**0.006**	**0.007**	0.604	0.785	0.145	**0.000**	**0.000**	**0.000**	**0.000**	**0.000**	**0.000**	**0.000**
	Louru	**0.010**	0.839	**0.006**	**0.037**	0.112	0.927	0.086	**0.000**	**0.000**	**0.000**	**0.000**	**0.000**	**0.002**	**0.001**
	Tornillo	0.256	0.093	**0.005**	0.157	0.927	0.969	0.785	**0.016**	**0.000**	**0.000**	**0.000**	**0.000**	**0.000**	**0.000**
	Cumala tree	0.255	0.061	**0.003**	0.205	0.696	0.604	0.112	**0.000**	**0.000**	**0.000**	**0.000**	**0.000**	**0.000**	**0.000**
	Kapok	0.291	**0.015**	**0.001**	**0.008**	0.356	0.086	**0.026**	**0.000**	**0.000**	**0.000**	**0.000**	**0.000**	**0.004**	**0.029**
Fish:	Fish index (z-score)	n.a.	n.a.	n.a.	n.a.	0.927	0.515	0.185	0.763	**0.000**	**0.002**	**0.008**	**0.010**	**0.004**	0.250
	Arapaima	0.475	**0.012**	**0.000**	**0.024**	0.927	0.515	0.233	**0.012**	**0.000**	**0.000**	**0.001**	**0.002**	**0.008**	0.071
	Tambaqui	0.654	**0.003**	**0.001**	**0.014**	0.233	0.233	**0.019**	**0.018**	**0.001**	**0.002**	**0.005**	**0.013**	**0.006**	0.334
	Red-bellied pacu	0.994	**0.013**	**0.013**	**0.012**	0.290	0.112	0.145	**0.005**	**0.011**	**0.040**	0.206	0.075	0.088	0.530
	Peacock bass	0.234	**0.015**	**0.001**	**0.021**	0.515	0.086	0.112	0.056	**0.015**	**0.007**	0.101	0.070	**0.000**	0.134
	Wolf fish	0.641	0.133	0.268	0.063	0.969	0.515	0.927	0.763	0.676	0.844	0.643	0.314	0.705	0.421

P-values for t test for the equality of means of ILK measures between respondent types reported in Fig. 2 are in columns 1-4; p-values for Kolmogorov-Smirnov (K-S) test for the equality of distributions of ILK measures between respondent types reported in Figs. S6 and S7 are in columns 5-8; and p-values for correlations between ILK and land cover measures in each respondent type reported in Fig. 3 are in columns 9-14. p-values smaller than 0.05 are bolded.

Table S4. P-values for t test and correlations by cooperative forest clearing

		t test in Fig. S15		Correlation in Fig. 4			
Cooperative forest clearing		Low intensity	High intensity	Low intensity		High intensity	
Respondent type		Users vs. nonusers	Users vs. nonusers	Users	Nonusers	Users	Nonusers
		(1)	(2)	(3)	(4)	(5)	(6)
Game:	Game index (z-score)	n.a.	n.a.	**0.020**	**0.001**	**0.031**	**0.014**
	Lowland tapir	**0.001**	**0.000**	**0.017**	**0.003**	0.128	**0.034**
	White-lipped peccary	**0.014**	**0.000**	**0.063**	**0.006**	**0.006**	**0.001**
	Collared peccary	**0.000**	**0.000**	0.141	**0.038**	0.622	0.612
	Capybara	0.055	0.194	0.805	0.893	**0.015**	0.364
	Paca	0.102	**0.004**	0.074	**0.048**	0.529	0.499
	Brocket deer	**0.001**	**0.000**	**0.020**	**0.006**	0.368	0.345
	Red howler monkey	**0.019**	**0.003**	0.481	**0.026**	0.313	0.082
	Woolly monkey	0.068	**0.000**	**0.016**	**0.003**	**0.003**	**0.000**
	Spider monkey	0.404	**0.006**	0.195	0.130	**0.002**	**0.008**
Timber:	Timber index (z-score)	n.a.	n.a.	**0.000**	**0.000**	**0.000**	**0.000**
	Mahogany	0.393	0.059	0.281	0.270	0.162	0.625
	Spanish cedar	0.760	**0.001**	**0.005**	0.155	**0.010**	**0.000**
	Louru	0.940	**0.006**	0.360	0.251	**0.012**	**0.001**
	Tornillo	0.652	0.174	**0.000**	**0.001**	**0.000**	**0.001**
	Cumala tree	0.845	**0.046**	**0.001**	**0.002**	**0.000**	**0.000**
	Kapok	0.949	**0.000**	0.460	0.300	**0.045**	0.086
Fish:	Fish index (z-score)	n.a.	n.a.	0.426	0.704	**0.000**	0.068
	Arapaima	0.421	**0.021**	0.864	0.840	**0.001**	**0.021**
	Tambaqui	0.226	**0.029**	0.352	0.572	**0.006**	0.084
	Red-bellied pacu	0.720	**0.000**	0.860	0.116	**0.011**	0.735
	Peacock bass	0.490	**0.012**	**0.012**	0.583	**0.014**	0.162
	Wolf fish	0.877	**0.024**	0.759	0.810	0.315	0.237

P-values for t test for the equality of means of ILK measures between respondent types reported in Fig. S15 are in columns 1 and 2; and p-values for correlations between ILK and land cover measures in each respondent type reported in Fig. 4 are in columns 3-6. p-values smaller than 0.05 are bolded.
